# SUMO modification of a heterochromatin histone demethylase JMJD2A enables viral gene transactivation and viral replication

**DOI:** 10.1371/journal.ppat.1006216

**Published:** 2017-02-17

**Authors:** Wan-Shan Yang, Mel Campbell, Pei-Ching Chang

**Affiliations:** 1 Institute of Microbiology and Immunology, National Yang-Ming University, Taipei, Taiwan, R.O.C.; 2 UC Davis Cancer Center, University of California, Davis, Davis, California, United States of America; 3 Center for Infectious Disease and Cancer Research, Kaohsiung Medical University, Kaohsiung, Taiwan, Republic of China; University of Southern California, UNITED STATES

## Abstract

Small ubiquitin-like modifier (SUMO) modification of chromatin has profound effects on transcription regulation. By using Kaposi’s sarcoma associated herpesvirus (KSHV) as a model, we recently demonstrated that epigenetic modification of viral chromatin by SUMO-2/3 is involved in regulating gene expression and viral reactivation. However, how this modification orchestrates transcription reprogramming through targeting histone modifying enzymes remains largely unknown. Here we show that JMJD2A, the first identified Jumonji C domain-containing histone demethylase, is the histone demethylase responsible for SUMO-2/3 enrichment on the KSHV genome during viral reactivation. Using *in vitro* and *in viv*o SUMOylation assays, we found that JMJD2A is SUMOylated on lysine 471 by KSHV K-bZIP, a viral SUMO-2/3-specific E3 ligase, in a SUMO-interacting motif (SIM)-dependent manner. SUMOylation is required for stabilizing chromatin association and gene transactivation by JMJD2A. These finding suggest that SUMO-2/3 modification plays an essential role in the epigenetic regulatory function of JMJD2A. Consistently, hierarchical clustering analysis of RNA-seq data showed that a SUMO-deficient mutant of JMJD2A was more closely related to JMJD2A knockdown than to wild-type. Our previous report demonstrated that JMJD2A coated and maintained the “ready to activate” status of the viral genome. Consistent with our previous report, a SUMO-deficient mutant of JMJD2A reduced viral gene expression and virion production. Importantly, JMJD2A has been implicated as an oncogene in various cancers by regulating proliferation. We therefore further analyzed the role of SUMO modification of JMJD2A in regulating cell proliferation. Interestingly, the SUMO-deficient mutant of JMJD2A failed to rescue the proliferation defect of JMJD2A knockdown cells. Emerging specific inhibitors of JMJD2A have been generated for evaluation in cancer studies. Our results revealed that SUMO conjugation mediates an epigenetic regulatory function of JMJD2A and suggests that inhibiting JMJD2A SUMOylation may be a novel avenue for anti-cancer therapy.

## Author summary

Epigenetic dysregulation connects genotype to diseases. An understanding of epigenetic regulation holds promise for clinical use. The profound epigenetic changes that occur during the latent-to-lytic switch of the Kaposi’s sarcoma associated herpesvirus (KSHV) life cycle make it an attractive model system for studies of epigenetic regulation. Using this model, our recent work showed that the demethylase JMJD2A and SUMO-2/3 specific modifications of viral and host chromatin are associated with epigenetic regulation of transcription during reactivation. However, how SUMO modification and histone modifying enzymes interface to orchestrate epigenetic regulation remains largely unknown. Here, we demonstrate JMJD2A as an example of a histone demethylase that is SUMO-2/3 modified by the KSHV encoded SUMO E3 ligase, K-bZIP. SUMO modification of JMJD2A is essential for stabilizing its chromatin binding and exerting its transcriptional derepression activity. Emerging evidence has implicated JMJD2A as an oncogene involved in the progression of various human tumors. The essential role of SUMO in regulating the biological function of JMJD2A suggests that SUMOylation of JMJD2A may be one of the potential underlying mechanisms responsible for JMJD2A-mediated oncogenesis. In this regard, inhibition of JMJD2A SUMOylation could be a new strategy for anti-cancer therapy.

## Introduction

Epigenetics connects genotype to phenotype and disease. Histone modifications are important epigenetic events in the regulation of chromatin structure and gene transcription. Different from acetylation, histone lysine methylation was long considered to be irreversible and therefore garnered little attention in the epigenetics field until the discovery of first histone lysine demethylase (KDM), LSD1/KDM1A [1]. LSD1 catalyzes a flavin-dependent amine oxidation reaction that can only demethylate mono- and di-methylated forms of modified histone lysine residues. Removal of trimethyl groups from histone lysines was first evidenced by the discovery of Jumonji C (JmjC) domain-containing histone demethylase 2A (JMJD2A) in 2006 [2]. This enzyme catalyzes a Fe(II) and α-ketoglutarate-dependent dioxygenation reaction that demethylates the tri-methyl group on modified histone lysine residues. The realization that all histone lysine methylation states are completely reversible established histone methylation as a novel component of the “histone code” for epigenetic regulation. It is therefore not surprising that intensive studies have focused on the role of JMJD2A in epigenetics and disease regulation.

JMJD2A (also known as KDM4A and JHDM3A) is a histone 3 lysine 9 (H3K9) and lysine 36 (H3K36) trimethyl-specific KDM [2] with specificity towards trimethyl H3K9 (H3K9me3) [3]. Similar to all KDMs, an intact JmjC domain is required for demethylation activity of JMJD2A and a single amino acid mutation on histidine 188 (H188) within JmjC domain completely abolishes demethylation activity [2]. In addition, JMJD2A possess a double tudor domain at its C-terminus [4]. This domain binds to H3K4me3 and H4K20me2/3 and functions as a chromatin recruitment binding module [4,5]. However, the target specificity of JMJD2A also depends on its interacting partners. For example, interaction with HP1 enhances H3K36me3 demethylation activity of JMJD2A [6,7]. Although considerable progress has been made in understanding the specificity of JMJD2A in binding to and demethylation of histone marks, regulation of JMJD2A-chromatin interactions are largely unknown and requires further analysis.

Post-translational modifications (PTMs) play important roles in determining protein function. Increasing evidence points to PTM regulation of the functional specificity of KDMs. For example, phosphorylation of JmjC domain-containing histone demethylase PHD finger protein 2 (PHF2) enables PHF2 activation and subsequent formation of a histone demethylase complex that specifically binds to its target promoters and demethylates H3K9me2 [8]. Similarly, phosphorylation of KDM3A determined its specific binding to target genes [9]. In contrast, phosphorylation of LSD1 dissociates corepressors from its histone demethylase complex and consequently impairs the repressive activity of LSD1 [10]. In addition to phosphorylation, modification of KDMs by other PTMs, such as ubiquitination and SUMOylation, has also been reported. Ubiquitination of JMJD2A, KDM4B, KDM5C and PHF8 invariably mediates proteasome-dependent degradation of these KDMs [11–14]. SUMOylation of KDM5B, a transcription repressor that demethylates the activation mark H3K4me3, prevents its occupancy at target genes [15]. However, little is known about small ubiquitin-like modifier (SUMO) modification in regulating other KDMs.

SUMO was initially identified as a reversible PTM that regulates protein stability and signal transduction [16–18]. The growing list of SUMO modified DNA binding proteins or transcription factors reveals the importance of SUMOylation in chromatin remodeling complex formation and transcription regulation [16]. Genome-wide studies have shown that SUMO modification may associate with either positive regulation [19,20] or negative restraint of gene transcription [20–22]. By using Kaposi’s sarcoma associated herpesvirus (KSHV) as a model, we recently showed that SUMO-2/3 specific chromatin modification restrains the transactivation of genes in the KSHV genomic euchromatin region [21,23]. This suggests that SUMO-paralog specific chromatin modifications may be involved in the observed variation in the role of SUMO in epigenetic regulation of transcription. However, the SUMO-2/3 target proteins on the KSHV euchromatin region have never been identified. Uncovering the target proteins will not only largely improve our knowledge of SUMO modification in epigenetic regulation but also open an avenue for developing specific inhibitors for therapeutic use.

Following our previous report showing the JMJD2A binding profile in the euchromatin region of KSHV latent genomes [24], we first examined the essentialness of JMJD2A for SUMO-2/3 enrichment in the KSHV genome euchromatin regions upon reactivation. Our results revealed a striking reduction of SUMO-2/3 enrichment in JMJD2A knockdown cells when compared with control cells. We found that JMJD2A is modified by SUMO-2/3 at K471 and that this modification is important for JMJD2A binding on viral chromatin and for viral gene transactivation during KSHV reactivation. Moreover, we identified that KSHV lytic protein K-bZIP, a viral SUMO E3 ligase with specificity towards SUMO-2/3 [25], enhances SUMOylation of JMJD2A. Emerging evidence has underscored the association of JMJD2A activity with various cancers (reviewed in [26]). Interestingly, we found that wild-type (WT) but not SUMO-deficient mutant of JMJD2A was capable of rescuing the proliferation of JMJD2A knockdown cells. Cellular genes that are highly upregulated in JMJD2A-WT during KSHV reactivation are enriched in cancer-related pathways when compared against the SUMO deficient mutant. We also show that JMJD2A binds at the promoter region of the oncogenic *TBX3* and that SUMOylation of JMJD2A is required for its full *TBX3* induction. Oncogenic viruses have served as important experimental models to identify oncogenes and study the molecular mechanisms underlying oncogenesis. These findings provide some clues to understand potential mechanisms underlying tumorigenesis mediated by JMJD2A. Therapeutic inhibition of JMJD2A has been implicated as a potential target in cancer therapy. Since SUMOylation is essential for JMJD2A binding to target gene promoters and executing its epigenetic function, inhibiting JMJD2A SUMOylation could be a new strategy for cancer therapy.

## Results

### JMJD2A is required for efficient SUMO-2/3 enrichment on KSHV genome

**Fig 1:**
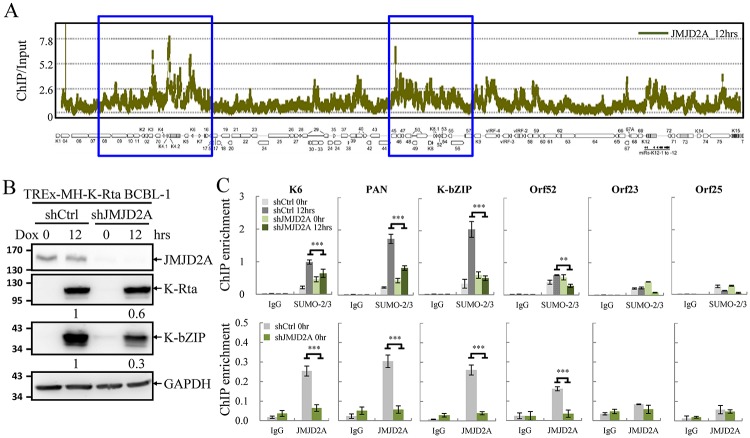
JMJD2A is required for efficient SUMO-2/3 enrichment on the viral genome during KSHV reactivation. (A) ChIP-on-chip analysis of JMJD2A binding across the KSHV lytic genome. A JMJD2A ChIP assay was performed on TREx-MH-K-Rta BCBL-1 cells treated with 0.2 μg/ml doxycycline (Dox) for 12 hours (hrs). The genomic locations of KSHV ORFs are depicted below the histogram. The blue rectangular areas are locations that comprise high SUMO-2/3 levels after KSHV reactivation (Yang et al. 2015). (B) Total cell lysates (TCLs) from TREx-MH-K-Rta-shCtrl and -shJMJD2A BCBL-1 cells before and after Dox treatment (12 hrs) were immunoblotted with antibodies as indicated. Ratio is the relative signal of K-Rta or K-bZIP to GAPDH observed for Dox treatment at 12 hrs using TREx-MH-K-Rta-shCtrl BCBL-1 cells set as 1.0. (C) ChIP was performed with chromatin prepared from cells treated as described in (B) using rabbit IgG, anti-SUMO-2/3 (upper panel) and anti-JMJD2A (lower panel) antibodies. ChIP DNA was quantified by real-time quantitative PCR (qPCR) using primer pairs specific for promoter regions of KSHV *K6*, *PAN*, *K-bZIP*, *Orf52*, *Orf23* and *Orf25*. (Data represent mean±SEM. n = 3. **p<0.01. ***p<0.005).

We have previously reported a global SUMO-2/3 enrichment on KSHV genome euchromatin regions upon viral reactivation [23]. In this study we sought to identify potential SUMO targets residing on viral chromatin. The negative correlation between SUMO-2/3 enrichment and the heterochromatin mark H3K9me3 in the KSHV lytic genome [23] is reminiscent of the inverse correlation between H3K9me3 with JMJD2A in latent viral chromatin that we reported in 2011 [24]. Moreover, in the same report, we demonstrated that the KSHV SUMO E3 ligase K-bZIP interacts with JMJD2A and inhibits its demethylase activity. Together, these results suggest that JMJD2A may be a potential SUMO target on viral chromatin. To study this, we first performed a chromatin immunoprecipitation (ChIP) experiment of JMJD2A using chromatin prepared from TREx-F3H3-K-Rta BCBL-1 cells after doxycycline (Dox) induction for 12, 24, and 36 hours (S1 Fig). A KSHV tiling array [24] was then used to measure the binding of JMJD2A on viral lytic chromatin at 12 hours post induction. The ChIP-on-chip result revealed a comparable binding pattern of JMJD2A throughout the KSHV lytic (Fig 1A) and latent (data published in Fig 4A of J. Virol., 2011 [24]) genome. Pearson correlation showed a strong positive relationship between JMJD2A binding on the KSHV latent and lytic genome (r = 0.83) as expected. However, Pearson's analysis showed no statistically significant correlation between global SUMO-2/3 enrichment (Yang et al. 2015) and JMJD2A binding (r = 0.21) on the KSHV genome at 12 hours post induction. These data suggest that instead of being a genome-wide target for SUMO conjugation, JMJD2A may function in a locus-specific manner.

To identify potential JMJD2A binding loci that may responsible for SUMO-2/3 enrichment on KSHV genome during reactivation, we aligned the ChIP-seq data of SUMO-2/3 [23] and ChIP-on-chip data of JMJD2A (Fig 1A) on the KSHV lytic genome. We noticed that two viral genome regions, which contain high levels of JMJD2A binding, also displayed a significant increase of SUMO-2/3 (Fig 1A, blue boxes). This finding indicates that JMJD2A may be the SUMO target in these two KSHV genome regions and responsible for the SUMO-2/3 enrichment during viral reactivation. If this is true, loss of JMJD2A will abolish the SUMO-2/3 enrichment. To study this, we performed another ChIP assay of SUMO-2/3 and JMJD2A using chromatin prepared from JMJD2A knockdown TREx-MH-K-Rta-shJMJD2A BCBL-1 and its control cells (TREx-MH-K-Rta-shCtrl BCBL-1) (Fig 1B). KSHV *K6* and *PAN* in the first region and *K-bZIP* and *Orf52* in the second region were chosen for quantitative PCR (qPCR) analysis. ChIP-qPCR results showed that JMJD2A knockdown significantly reduced but did not completely abolish SUMO-2/3 enrichment on the promoter regions of KSHV genes in both regions (Fig 1C, upper panel). *Orf23* and *Orf25* which reside in a low SUMO enrichment region were used as negative controls. The significant decrease of JMJD2A (Fig 1C, lower panel), which correlates with reduced SUMO-2/3 enrichment by knockdown of JMJD2A implies that the SUMO-2/3 enrichment could be due to JMJD2A SUMO-2/3 conjugation at the indicated KSHV genome regions.

### JMJD2A is SUMOylated at K471

**Fig 2:**
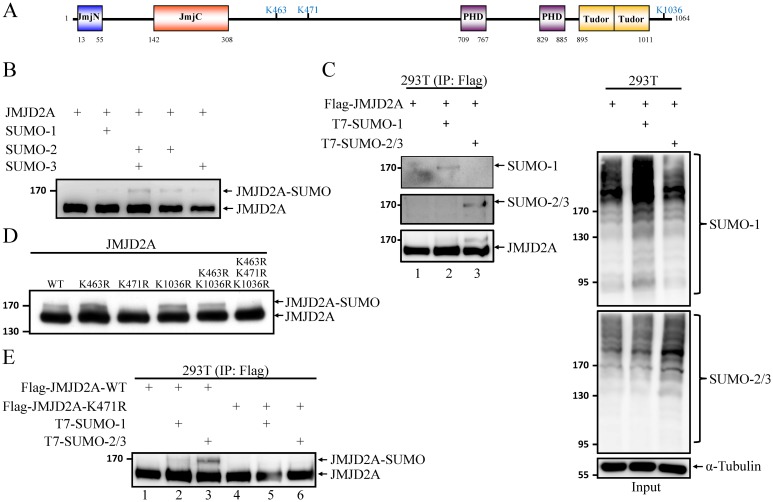
JMJD2A is SUMOylated at K471. (A) Schematic representation of the domain structure of JMJD2A and the three putative SUMOylation sites. (B) *in vitro* SUMOylation assay using recombinant Flag-JMJD2A and SUMO isoforms as substrates. (C) *in vivo* SUMOylation assay was performed by transfecting 293T cells with plasmids expressing Flag-JMJD2A (0.4 μg) and T7-SUMO-1 or T7-SUMO-2 and -3 (1.2 μg). JMJD2A was immunoprecipitated (IP’d) with anti-Flag-M2 beads and analyzed by immunoblotting using anti-SUMO-1, anti-SUMO-2/3 and anti-JMJD2A antibodies (left panel). TCLs were immunoblotted with anti-SUMO-1, anti-SUMO-2/3 antibodies and anti-α-Tubulin (right panel). (D) Purified wild-type (WT) and different combinations of SUMOylation site mutants of Flag-JMJD2A were used for *in vitro* SUMOylation assay as described in (B). 4%-20% gradient SDS-PAGE was used to resolve JMJD2A and SUMOylated JMJD2A. (E) Flag-JMJD2A-WT or -K471R was expressed in 293T cells as described in (C) and IP’d using M2 beads. SUMOylated JMJD2A was analyzed with anti-JMJD2A antibody.

To examine whether JMJD2A is post-translationally modified by SUMO, we first carried out a cell-free *in vitr*o SUMOylation assay using recombinant and purified SUMOylation E1 and E2 enzymes, the substrate JMJD2A and SUMO paralogs. A higher molecular weight band representing SUMO-modified JMJD2A was observed in reactions containing SUMO-2 and/or SUMO-3 with the highest level of SUMO modification in the presence of SUMO-2/3 (Fig 2B). SUMO modification of JMJD2A was further evaluated *in vivo*. To this end, we transiently transfected plasmids expressing Flag-tagged JMJD2A together with T7-tagged SUMO-1 or SUMO-2 and -3 in 293T cells and immunoprecipitated (IP’d) the total cell lysates (TCLs) with M2 affinity beads. Western blot analysis of TCLs using SUMO-1 and SUMO-2/3 antibodies showed the successful overexpression and conjugation of SUMO-1 and SUMO-2/3 in 293T cells (Fig 2C, right panel). Immunoblotting of the precipitated JMJD2A using anti-SUMO-1 and anti-SUMO-2/3 antibodies revealed that it was conjugated by both SUMO-1 and SUMO-2/3 (Fig 2C, left panel). Immunoblotting using anti-JMJD2A antibodies showed a lower level of conjugation with SUMO-1 (compare lanes 2 and 3 in Fig 2C, left panel). Following our discovery of JMJD2A SUMOylation, SUMOsp2.0 and SUMOplot Analysis Programs were used to predict potential SUMOylation site(s) on JMJD2A. By overlapping the sites predicted by both algorithms, we narrowed down the putative SUMO sites in JMJD2A to three lysines (K463, K471, and K1036) (Fig 2A). To determine whether JMJD2A is SUMOylated at these three putative SUMOylation sites, we generated a set of single- (K463R, K471R, and K1036R), double- (K463R/K1036R), and triple- (K463R/K471R/K1036R) site mutants of JMJD2A in which the lysine residues of the putative SUMOylation sites were replaced by arginine (R). *in vitr*o SUMOylation assays using purified wild-type (WT) and mutant JMJD2A proteins showed the disappearance of the main JMJD2A-SUMO band in the K471R and triple mutants (Fig 2D), indicating that K471 may contribute to the SUMO conjugation of JMJD2A.

To confirm this, we further evaluated the capability of the WT and K471R mutant JMJD2A proteins to be modified by SUMO *in vivo*. Consistently, SUMO modification of K471R mutant was undetectable (Fig 2E, compare lanes 1–3 versus lanes 4–6, respectively), confirming that K471 in JMJD2A is involved in the conjugation of SUMO to JMJD2A. Moreover, we transiently transfected plasmids expressing Flag-tagged JMJD2A-K463R/K1036R mutant together with T7-tagged SUMO-1 or SUMO-2 and -3 in 293T cells and immunoprecipitated (IP’d) the TCLs with M2 affinity beads. Western blot analysis of TCLs showed the successful overexpression and conjugation of SUMO-1 and SUMO-2/3 in 293T cells (S2A Fig). Immunoblotting of the precipitated JMJD2A using anti-JMJD2A, anti-SUMO-1 and anti-SUMO-2/3 antibodies revealed that JMJD2A-K463R/K1036R mutant was conjugated by both SUMO-1 and SUMO-2/3 (S2B Fig). These results provide the first evidence for JMJD2A as a new SUMO target protein.

### SUMOylation of JMJD2A is enhanced by viral SUMO E3 ligase K-bZIP

**Fig 3:**
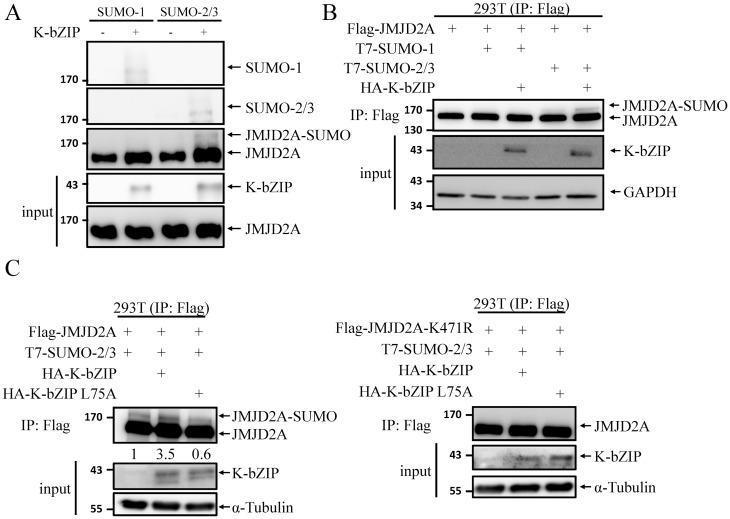
KSHV SUMO E3 ligase K-bZIP catalyses SUMO modification of JMJD2A. (A) *in vitro* SUMOylation assay of JMJD2A was performed with the indicated combination of SUMO isoforms and K-bZIP. JMJD2A and SUMOylated JMJD2A were resolved by 4%-20% gradient SDS-PAGE and immunoblotted using anti-SUMO-1, anti-SUMO-2/3 and anti-JMJD2A antibodies. Input of JMJD2A and K-bZIP was detected by immunoblotting using specific antibodies as indicated. (B) *in vivo* SUMOylation assay was performed as described in Fig 2C, with co-transfection of Flag-JMJD2A (0.4 μg), T7-SUMO-1 or T7-SUMO-2 and -3 (0.1 μg), and WT K-bZIP (0.1 μg). 4%-20% gradient SDS-PAGE was used to resolve JMJD2A and SUMOylated JMJD2A. Immunoblotting was performed using anti-JMJD2A, anti-K-bZIP and anti-GAPDH antibodies. (C) Flag-K-bZIP-WT or -L75A were co-expressed in 293T cells with Flag-JMJD2A and T7-SUMO-2 and -3 as described in (B). SUMOylated JMJD2A and input proteins were detected by immunoblotting using specific antibodies as indicated. Ratio is the relative signal of JMJD2A-SUMO to total JMJD2A observed using signal in lane 1 set as 1.0.

Following our previous report showing that JMJD2A colocalizes and interacts with K-bZIP [24], a SUMO-2/3 specific viral E3 ligase encoded by KSHV lytic *Orf K8* [25], we hypothesized that JMJD2A is a SUMOylation target of K-bZIP. To determine whether K-bZIP might enhance SUMOylation of JMJD2A, we first performed an *in vitr*o SUMOylation assays using purified K-bZIP. Immunoblotting using anti-SUMO-1, anti-SUMO-2/3 and anti-JMJD2A antibodies revealed that JMJD2A was SUMOylated by K-bZIP (Fig 3A). When SUMO E3 ligase dead mutant (L75A) of K-bZIP that we generated in our previous work [25] was included, we detected SUMOylated JMJD2A bands in WT but not the ligase activity dead mutant of K-bZIP (S2C Fig). This result indicates that K-bZIP promoted the SUMOylation of JMJD2A in an E3 ligase activity dependent manner. However, *in vitr*o SUMO-1 modification of JMJD2A was also observed in the presence of K-bZIP. This might due to the high protein level of SUMO and E3 ligase *in vitr*o that leads to the loss of SUMO paralog specificity. To confirm this, we further evaluated the K-bZIP-mediated SUMOylation of JMJD2A *in vivo*. We found that co-transfection of a plasmid expressing K-bZIP increased the SUMOylation of JMJD2A to a much higher level using SUMO-2/3 modification than with SUMO-1 (Fig 3B). This indicates that the SUMO-2/3 specificity of JMJD2A persists *in vivo*. The E3 ligase activity-dependent SUMOylation of JMJD2A by K-bZIP was further confirmed *in vivo*. As shown in Fig 3C, a significant increase of SUMO-2/3 modification of JMJD2A (left panel) but not its SUMOylation deficient K471R mutant (right panel) was observed in cells overexpressing WT K-bZIP but not its L75A mutant. These data indicate that JMJD2A is a novel SUMO substrate of viral SUMO E3 ligase K-bZIP.

### JMJD2A SUMOylation is essential for complete enrichment of SUMO-2/3 on KSHV genome during viral reactivation

**Fig 4:**
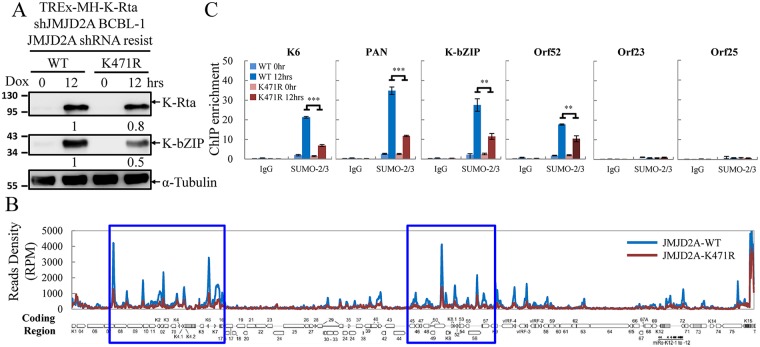
JMJD2A SUMOylation is crucial for SUMO-2/3 enrichment on the KSHV genome during viral reactivation. (A) TCLs from TREx-MH-K-Rta-shJMJD2A-Flag-JMJD2A-WT and -K471R BCBL-1 cells before and after 12 hrs Dox (0.2 μg/ml) treatment were immunoblotted with antibodies as indicated. Ratio is the relative signal of K-Rta or K-bZIP to α-Tubulin observed for Dox treatment at 24 hrs using TREx-MH-K-Rta-shJMJD2A-Flag-JMJD2A-WT BCBL-1 cells as 1.0. (B) ChIP-seq assay was performed with chromatin from cells treated as described in (A) using rabbit IgG and anti-SUMO-2/3 antibodies. Sequenced reads mapped to KSHV genome were normalized with total reads from the human genome. The histogram shows reads per million mapped reads (RPM) mapped across the KHSV genome (JMJD2A-WT, blue; -K471R, red). p = 2.27e-103 by *Student’s-t* test. The blue rectangles are high SUMO-2/3 regions as in Fig 1A. (C) ChIP-seq data was verified by real-time qPCR using primer pairs specific for promoter regions of KSHV *K6*, *PAN*, *K-bZIP*, *Orf52*, *Orf23* and *Orf25*. (Data represent mean±SEM. n = 3. **p<0.01. ***p<0.005).

To assess the functional significance of JMJD2A SUMOylation, we first generated rescue cell lines stably expressing WT and SUMO-deficient mutant (K471R) of JMJD2A in JMJD2A knockdown TREx-MH-K-Rta-shJMJD2A BCBL-1 cells. Immunoblotting confirmed the similar expression level of shRNA-resistant JMJD2A-WT and JMJD2A-K471R mutant to that of endogenous JMJD2A prior to knockdown (S3A Fig). Given that JMJD2A is required for efficient SUMO-2/3 enrichment on viral promoters during reactivation (Fig 1C), the essential contribution of K471 of JMJD2A in global SUMO-2/3 enrichment on the KSHV genome during reactivation was evaluated using ChIP-seq. The successful induction of K-Rta and expression of K-bZIP after Dox treatment for 12 hours were first confirmed by immunoblotting (Fig 4A). The ChIP experiments were then performed using chromatin prepared from the Dox-induced (lytic phase) TREx-MH-K-Rta-shJMJD2A-Flag-JMJD2A-WT and -K471R BCBL-1 cells. High-throughput sequencing was carried out to measure the chromatin binding of SUMO-2/3 following a ChIP assay. ChIP-seq data showed similar SUMO-2/3 distribution in JMJD2A-WT rescue cells when compared with TREx-F3H3-K-Rta BCBL-1 cells [23]. As shown in Fig 4B, the ChIP-seq result showed that the SUMO-2/3 enrichment was reduced on most parts of high JMJD2A binding regions in cells containing the K471R mutant of JMJD2A at 12 hours after viral reactivation (p = 2.27e-103). However, it should be noted that no significant changes of SUMO-2/3 binding was observed in several gene loci within high JMJD2A binding regions. Again, this result further supports the notion that SUMOylation of JMJD2A may function in a locus-specific manner. To further verify the ChIP-seq results, SUMO-2/3 binding on promoter regions of *K6*, *PAN*, *K-bZIP* and *Orf52* was analyzed using real-time qPCR. *Orf23* and *Orf25* in a low SUMO enrichment region were used as negative controls. Consistent with the ChIP-seq results, qPCR data showed that the genes in high JMJD2A binding regions tested here displayed a significant reduction of SUMO-2/3 enrichment after viral reactivation in cells containing JMJD2A-K471R when compared with WT control (Fig 4C). These results suggest that K471 is the primary SUMOylation site of JMJD2A that is responsible for at least part of SUMO-2/3 conjugation on KSHV chromatin during KSHV reactivation.

### SUMOylation-deficient JMJD2A mutant cells show diminished expression of viral genes and reduced virion production

**Fig 5:**
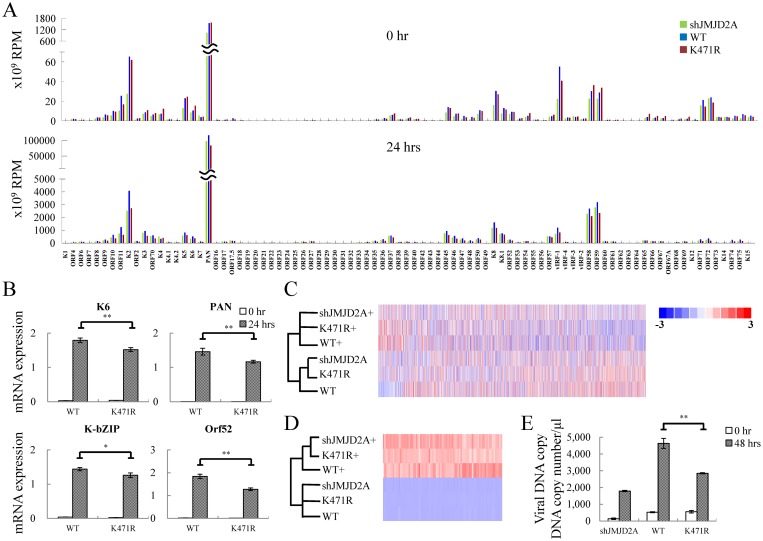
JMJD2A SUMOylation plays an essential role in KSHV viral gene transactivation and viral reactivation. (A) RNA-seq was performed using total RNA from non-induced (0 hrs, upper panel) and 0.2 μg/ml Dox treated (24 hrs, lower panel) TREx-MH-K-Rta-shJMJD2A (green bars), -shJMJD2A-Flag-JMJD2A-WT (blue bars) and -K471R (red bars) BCBL-1 cells. One representative RNA-seq expression dataset of KSHV genes is presented as reads per million (RPM) mapped. (B) RT-qPCR verification of KSHV *K6*, *PAN*, *K-bZIP* and *Orf52* expression in TREx-MH-K-Rta-shJMJD2A-Flag-JMJD2A-WT and -K471R BCBL-1 cells. (C and D) Heat map depicts hierarchical clustering of RNA-seq data of cellular (C) and KSHV (D) gene expression. (+, plus Dox 24h). (E) Supernatants from TREx-MH-K-Rta-shJMJD2A, -shJMJD2A-Flag-JMJD2A-WT and -K471R BCBL-1 cells treated as described in (A) for 48 hrs were collected, filtered, and the viral titers were determined by analyzing the virion-associated DNA levels using TaqMan qPCR. (Data represent mean±SEM. n = 3. **p<0.01.)

Our previous report showed that SUMO-2/3 enrichment on the KSHV lytic genome restrains viral gene expression [23]. We therefore hypothesized that SUMOylation of K471 in JMJD2A may also participate in restraining viral lytic gene expression and that loss of SUMOylation in the K471R mutant may result in higher viral gene expression during reactivation. To explore this idea, an RNA-seq assay was performed using total RNA purified from JMJD2A knockdown BCBL-1 cells and rescue cell lines stably expressing WT and K471R of JMJD2A before and after K-Rta-induced reactivation. The expression of JMJD2A, K-Rta and K-bZIP before and after Dox treatment for 24 hours was shown by immunoblotting (S3B Fig). Consistent with our previous report showing that JMJD2A is essential for maintaining the readiness of KSHV genes to be reactivated [24], when compared with JMJD2A-WT rescue cells (blue in Fig 5A), knockdown of JMJD2A reduced viral gene activation during reactivation (green in Fig 5A). Surprisingly, instead of eliciting a higher induction of viral genes expression, the rescue cell line expressing JMJD2A-K471R showed a lower activation of viral genes at 24 hours after reactivation (red in Fig 5A) when compared with WT control (blue in Fig 5A). To validate gene expression changes observed in RNA-seq analysis, expression of *K6*, *PAN*, *K-bZIP* and *Orf52* before and after viral reactivation was analyzed using real-time reverse transcription-qPCR (RT-qPCR). Consistent with RNA-seq results, a slightly, but statistically significant, diminished expression of these viral genes expression was observed during lytic reactivation in JMJD2A-K471R (Fig 5B).

Interestingly, the data suggests that JMJD2A-K471R mimics the knockdown of JMJD2A. To verify this observation, we performed a hierarchical clustering analysis using the RNA-seq results of both cellular (Fig 5C) and viral (Fig 5D) datasets across the panel of JMJD2A knockdown, JMJD2A WT and K471R cells. The result showed that in both datasets the latent cells align at closer distances than lytic cells. For viral genes after reactivation, the aligned distances of WT were far from both knockdown and K471R of JMJD2A (Fig 5D). This result supports the notion that JMJD2A-K471R may function similar to its knockdown. Since our previous report showed that knockdown of JMJD2A reduced virion production [24], viral titers were determined in TREx-MH-K-Rta-shJMJD2A, -shJMJD2A-Flag-JMJD2A-WT and -K471R BCBL-1 cells at 48 hours with or without Dox treatment. Immunoblotting showed the successful induction of K-Rta and expression of KSHV lytic protein K-bZIP (S3C Fig). KSHV virion-associated DNA was purified from viral particles in supernatants and determined by real-time qPCR as previously described [24]. Similar to the knockdown, the JMJD2A-K471R significantly reduced viral production when compared with JMJD2A-WT rescue cell lines (Fig 5E).

### SUMOylation modulates the chromatin binding and histone demethylase activity of JMJD2A

**Fig 6:**
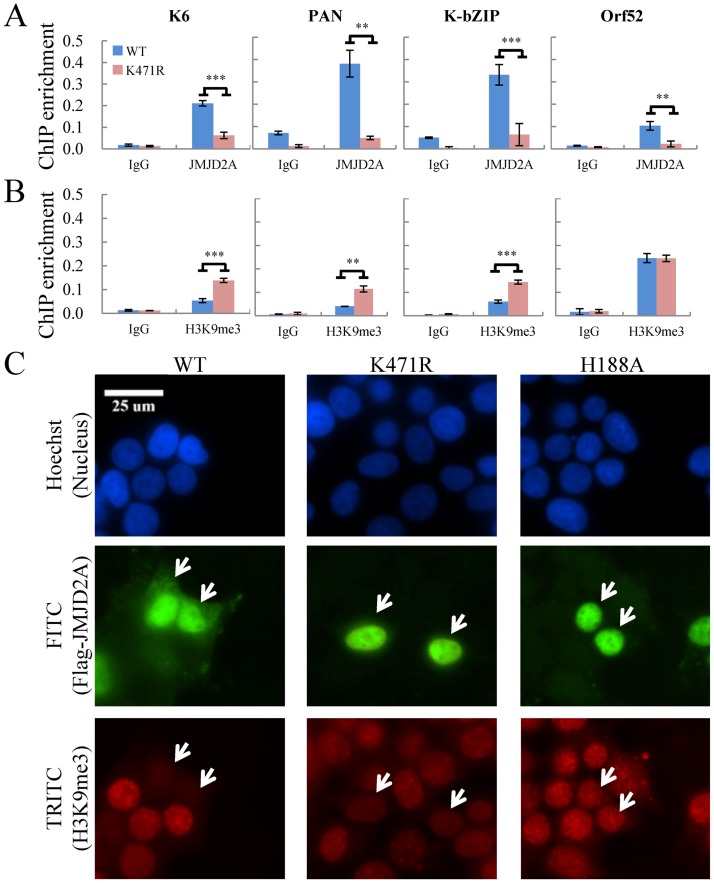
SUMOylation modulates the occupancy and demethylase activity of JMJD2A at target genes. (A and B) ChIP was performed with chromatin prepared from TREx-MH-K-Rta-shJMJD2A-Flag-JMJD2A-WT and -K471R BCBL-1 cells using rabbit IgG, anti-JMJD2A (A) and anti-H3K9me3 (B) antibodies. ChIP DNA was quantified as described in Fig 1C. (C) Histone demethylase activity of Flag-tagged JMJD2A-WT, -K471R or -H188A protein in 293T cells was assessed by immunofluorescence staining (IF) using deconvolution fluorescence microscopy. Cells were fixed, stained with antibody specific for Flag (FITC) and H3K9me3 (TRITC), and mounted in Slow Fade Gold with DAPI. White arrows indicate cells transfected with Flag-JMJD2A-WT or its mutant.

SUMOylation has been recognized as a PTM that regulates gene transcription through modulating DNA-binding of chromatin-associated factors [27]. However, functional responses vary among target proteins, as DNA-binding activity may be positively or negatively modulated by SUMO. Our data imply SUMOylation may help stabilize JMJD2A binding on chromatin. To study this, we performed a ChIP in TREx-MH-K-Rta-shJMJD2A-Flag-JMJD2A-WT and -K471R BCBL-1 cells using anti-JMJD2A antibody. Consistent with our hypothesis, JMJD2A-K471R significantly reduced its association with viral chromatin (Fig 6A). In addition, ChIP analysis using anti-H3K9me3 antibody demonstrated significantly higher levels of H3K9me3 in JMJD2A-K471R at three (*K6*, *PAN*, *K-bZIP*) out of four viral promoter regions analyzed (Fig 6B). These findings suggest that SUMOylation at K471 is essential for the association of JMJD2A with chromatin and in mediating the demethylation of H3K9me3. To further confirm that the histone demethylase activity of JMJD2A is regulated by SUMOylation, we examined H3K9me3 levels in 293T cells transiently transfected JMJD2A-WT or -K471R. The catalytically deficient JMJD2A-H188A mutant was also included as a negative control. Forty-eight hours after transfection, cells were fixed and co-stained with antibodies against JMJD2A and H3K9me3. Consistent with previous reports, overexpression JMJD2A, but not its catalytically deficient mutant, diminished H3K9me3 levels (Fig 6C, left and right panel, respectively). Immunostaining showed similar H3K9me3 intensity in K471R overexpressing cells versus non-transfected cells (Fig 6C, middle panel). This was further confirmed by *in vitro* demethylation assay. Flag-tagged WT or K471R JMJD2A proteins purified from Sf9 cells (S4A Fig) or IP’d from 293T cells (S4B Fig) were incubated with histone proteins in demethylase buffer. Consistently, immunoblotting results showed that JMJD2A-K471R failed to reduce H3K9me3 levels (S4 Fig). Taken together, these results imply that SUMOylation is important for chromatin binding and histone demethylation activity of JMJD2A.

### SUMOylation of JMJD2A and regulation of cellular gene expression during KSHV reactivation

**Fig 7:**
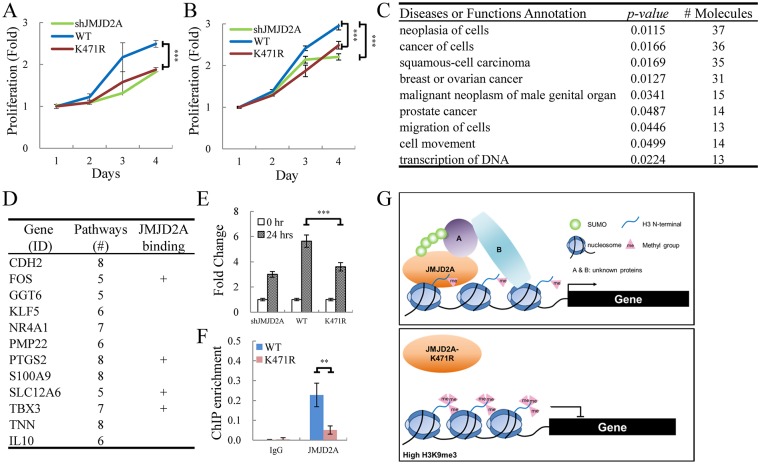
JMJD2A SUMOylation affects the expression of cellular genes involved in cancer. (A and B) WT JMJD2A but not its K471R mutant rescue proliferation of JMJD2A knockdown SLK (A) and BCBL-1 (B) cells. (A) SLK cells were sequentially infected with lentivirus expressing JMJD2A shRNA and Flag-tagged WT or K471R of JMJD2A. Cell proliferation was assessed by MTT assay. (B) Proliferation of TREx-MH-K-Rta-shJMJD2A, shJMJD2A-Flag-JMJD2A-WT and -K471R BCBL-1 cell lines was assessed by MTT assay. (C) Gene function analysis of cellular genes less upregulated in SUMOylation-deficient JMJD2A mutant (K471R) compared to JMJD2A-WT 24 hrs after KSHV reactivation. Gene list was shown in S7 Table. (D) Genes present in more than five identified functional pathways in (C). The plus (+) indicates genes with JMJD2A binding on the promoter region (transcription start site (TSS) ± 500bp). Gene list was shown in S8 Table. (E) RT-qPCR verification of *TBX3* expression in TREx-MH-K-Rta-shJMJD2A, -shJMJD2A-Flag-JMJD2A-WT and -K471R BCBL-1 cells. (F) qPCR was performed with ChIP DNA from Fig 6A using primer pairs specific for promoter region of *TBX3* (G) Schematic diagram illustrates the SUMO modification of JMJD2A in regulation gene transcription during KSHV reactivation.

JMJD2A was recently proposed as an oncoprotein [28,29]. Targeting JMJD2A by a KSHV lytic protein may contribute to the essential role of lytic phase in KSHV oncogenesis. To gain a better understanding of JMJD2A SUMOylation in modulating the host response to KSHV, we first compared the proliferation rate of control and transient JMJD2A knock down SLK cells (S6A Fig). Consistent with previous reports [30,31], knockdown JMJD2A significantly reduced the cell proliferation (S6B Fig). Only WT JMJD2A but not its K471R mutant could rescue the cell proliferation defect in JMJD2A knockdown SLK cells (Fig 7A). Similar result was observed in JMJD2A knockdown and its WT and K471R mutant rescued BCBL-1 cell lines (Fig 7B). These data indicate that SUMOylation is important for JMJD2A regulation of cell proliferation.

Consistent with our earlier report showing that binding of JMJD2A on viral chromatin maintains an open state for rapid activation of KSHV genes [24], our data here showed that SUMOylation of JMJD2A is required for maintaining its binding on the viral genome and an open chromatin structure for viral gene transactivation (Figs 6 and 5, respectively). Next, we asked whether this was also the case for the JMJD2A regulation of cellular genes. To this end, we first dissected the genes that are up-regulated in TREx-MH-K-Rta-shJMJD2A-Flag-JMJD2A-WT BCBL-1 cells during reactivation (628 genes) into three categories: 1.5-fold higher up-regulated (253 genes), 1.5-fold less up-regulated (49 genes) and no change (326 genes) when compared with K471R mutant rescued cells. These data indicate that around half (52%) of the cellular genes up-regulated during KSHV reactivation are independent of JMJD2A status. Consistent with our hypothesis that SUMOylation of JMJD2A maintains an open chromatin configuration for gene transactivation, JMJD2A SUMOylation is important for successful up-regulation of cellular genes (40%) during KSHV reactivation.

To identify functional pathways involved in SUMOylation-mediated JMJD2A upregulation of cellular genes during KSHV reactivation, we performed an ingenuity pathway analysis (IPA) using the 253 genes that are upregulated in TREx-MH-K-Rta-shJMJD2A-Flag-JMJD2A-WT BCBL-1 cell when compared with the K471R mutant. Gene ontology (GO) analysis identified nine significant pathways (*p*<0.05) containing more than ten gene molecules (Fig 7C). Consistent with emerging evidence showing that JMJD2A is involved in various cancers [26], most of the pathways identified were cancer-related. To narrow down the genes for further validation, genes simultaneously present in more than five of the nine identified pathways were first selected (Fig 7D). We then use ChIP-seq analysis to uncover the direct binding of JMJD2A on the promoter region (500 bp up- and downstream of the transcription start site (TSS)) of the identified genes (S5 Fig). Significant binding of JMJD2A was defined as any two peaks height of at least 3 reads per million (RPM) within each promoter region. By using this cut-off, we identified four of the twelve genes with JMJD2A-WT but not its K471R mutant binding on their promoter region (Fig 7D). Interestingly, TBX3 is a T-box transcription factor that has been implicated in a wide range of carcinomas [32]) and in regulation of proliferation [31,32]. Significant higher induction of *TBX3* mRNA levels in JMJD2A-WT rescued cells in comparison with JMJD2A knockdown and K471R mutant cells during KSHV reactivation was confirmed by RT-qPCR (Fig 7E). In addition, the direct binding of JMJD2A-WT but not K471R mutant on *TBX3* promoter region was verified by ChIP-qPCR (Fig 7F). Our data here indicate that *TBX3* may be a novel JMJD2A target gene that is responsible for JMJD2A-mediated cell proliferation during KSHV reactivation. However, more detailed analysis is required to elucidate the pathological consequences of *TBX3* up-regulation by JMJD2A.

## Discussion

SUMO modification has emerged as an important PTM that regulates protein subcellular localization, stability, protein-protein interaction, and DNA binding [33]. In recent years, increasing evidence showed that covalent modification of histone modification enzymes by SUMO regulates chromatin organization and gene expression. For histone deacetylases (HDACs), SUMOylation increases their transcriptional repression and HDAC activities [34,35]. In contrast, SUMO modification of KDM5B reduced its DNA binding and transrepression activities [15]. Mammalian cells contain three protein-conjugating isoforms of SUMO: SUMO-1, and highly related SUMO-2 and SUMO-3 (SUMO-2/3). The varied effects of SUMO partly result from the isoforms of SUMO proteins conjugated to a given substrate. In this study, we identified SUMO-2/3 as the modifier of JMJD2A and showed that JMJD2A SUMOylation is required for its DNA binding and transcription derepression activities.

SUMO-2/3 is targeted to JMJD2A through a viral SUMO E3 ligase K-bZIP, an immediate-early protein encoded by KSHV K8. This event is SUMO-interacting motif (SIM)-dependent SUMOylation (S2C Fig) and allows a specific SUMO-2/3 modification to JMJD2A (Fig 3B and 3C). For E3 ligase-dependent SUMOylation, conjugation usually occurs at the lysine (K) residue within ψKxD/E consensus motif. Among the three lysine residues, K463, K471, and K1036, located within the consensus motif of JMJD2A, K471 was identified as being SUMO modified as mutation of this lysine to arginine (K471R) significantly reduced JMJD2A SUMOylation (Fig 2D and 2E). Consistent with our previous report showing that K-bZIP is a SUMO E3 ligase with specificity towards SUMO-2/3 [25] and required for SUMO-2/3 conjugation on KSHV genome euchromatin region during reactivation [23], we showed here that JMJD2A is a new SUMO substrate of K-bZIP and is responsible for SUMO-2/3 enrichment on KSHV genome euchromatin regions during viral reactivation (Fig 1C). However, since induction of K-bZIP is reduced in both JMJD2A knockdown (Fig 1B) and K471R mutant (Fig 4A), the reduction of this viral SUMO E3 ligase may also responsible for lower SUMOylation in viral promoters (Figs 1C and 4C). Moreover, since K471R mutant of JMJD2A significantly reduced but not completely abolished the SUMO-2/3 enrichment (Fig 4C), this suggests that there might be still additional SUMO target protein(s) present on the KSHV genome. K-bZIP, the viral SUMO E3 ligase that can interact with SUMO protein [25] and is SUMOylated [36] may belong to one of these unknown proteins.

Our recent report showed that SUMO-2/3 viral chromatin modification contributes to creation of a repressive environment that restrains viral gene expression during reactivation, as SUMO-2/3 knockdown increases the expression of KSHV lytic genes [23]. However, our data here demonstrated that the viral gene activation (Fig 5A and 5B) and virion production (Fig 5E) were decreased when the SUMOylation site on JMJD2A is mutated. This indicates that SUMOylation of JMJD2A activates instead of represses the viral gene expression during KSHV reactivation. SUMO modification of chromatin proteins provides a binding platform for protein complex formation, typically via a non-covalent SIM, and stabilizes their DNA binding as the case for the KAP-1 [37,38]. The inconsistency between these studies could be due to the essential role of SUMO modification in JMJD2A DNA binding, as our ChIP data show that JMJD2A-K471R loses its occupancy at promoter regions of KSHV genes (Fig 6A). This loss of binding may also explain why the SUMOylation site mutation of JMJD2A resulted in increased H3K9 methylation (Fig 6B and 6C). Loss of JMJD2A binding by the SUMO modification site mutant can therefore be viewed as loss of JMJD2A. This is further supported by hierarchical clustering analysis of the transcriptome data which showed that the aligned distance was closer in knockdown and K471R of JMJD2A (Fig 5C and 5D). Following this concept, the data here are consistent with our earlier finding that JMJD2A is important for maintaining a favorable chromatin structure to facilitate KSHV reactivation, as knockdown of JMJD2A decreased virion production [24]. We hypothesized that though JMJD2A can be viewed as a platform for SUMO-2/3 binding on chromatin, JMJD2A-K471R may mimic the knockdown of JMJD2A, but not of SUMO-2/3.

JMJD2A SUMOylation is worth further investigation since this modification may have other regulatory functions. For example, the stability of JMJD2A is regulated by ubiquitination [11,28,39]. Analogous to ubiquitination, SUMOylation is a multistep enzymatic modification of proteins at lysine residues. However, in contrast with ubiquitination, SUMOylation does not usually trigger degradation. Interestingly, we observed a gradual reduction of JMJD2A along with KSHV reactivation (S3B and S3C Fig and Fig 2B in [24]). Moreover, the JMJD2A-K471R was found to be degraded at an earlier time point following viral reactivation (S3B Fig). Our data suggest that SUMO-2/3 modification of JMJD2A by K-bZIP during KSHV reactivation helps stabilize JMJD2A from degradation. This hypothesis is consistent with one recent report showing that modification of HDAC1 by SUMO-1, but not by SUMO-2, facilitates its degradation [40]. However, more detailed analysis is required to further our understating of JMJD2A protein stability and its regulation by paralog-specific SUMO modification. In addition, little is known about how SUMOylation of JMJD2A is regulated by cellular E3 SUMO ligases. Differing from the ubiquitin pathway enzyme cascade which contains hundreds of E3 ligases, only several E3 ligases (PIAS family, RanBP2, and Pc2) have been identified so far for the SUMOylation machinery. Moreover, a SUMO E3 ligase, while increasing SUMOylation, is sometimes a requirement for SUMO-paralog specific conjugation. In particular, PIASy has recently been reported to mediate SUMO-2/3 specific conjugation of HDAC1 [40] and poly(ADP-ribose) polymerase 1 (PARP1) [41]. Our preliminary data showed that a member of the PIAS family, PIAS3, acts as specific E3 ligase that promotes SUMO-2/3 modification of JMJD2A (S7 Fig). However, we cannot exclude the possibility of the presence of other cellular SUMO E3 ligases that contribute to SUMOylation of JMJD2A. This question is worthwhile for detailed analysis in the future.

It can be imagined that JMJD2A, the first discovered tri-methyl histone demethylase responsible for removing both heterochromatin mark H3K9me3 and active chromatin mark H3K36me3 [2], should play important roles in various cellular functions, such as maintaining genome integrity and regulating gene transcription. Overexpression of JMJD2A induces copy gains on specific chromosomal domains [42] and degradation of JMJD2A is required for DNA repair by efficient recruitment of 53BP1 to DNA damage sites [43]. For transcription regulation, stabilized JMJD2A chromatin binding is a prerequisite for PPARγ-mediated transcription regulation of genes associated with lipogenesis [39]. In addition, JMJD2A is involved in regulating transcription of neural-specific genes and therefore required for neural stem cell differentiation [44]. Most interestingly, levels of JMJD2A were observed to change during cell cycle progression. Peaking in G1/S phase, JMJD2A antagonizes the occupancy of heterochromatin protein HP1γ, increases chromatin accessibility and consequently promotes S phase progression [13,45]. Due to the functional diversity of JMJD2A, it is not surprising that dysregulation of this histone modification “eraser” would contribute to tumor progression. In addition, virally induced JMJD2A SUMOylation appears to activate certain cellular oncogenic pathways, probably providing an explanation for the observation that viral lytic genes are always coexpressed with cellular cancer-associated pathways [46]. Indeed, accumulating studies have noted the overexpression of JMJD2A in various cancers (reviewed in [26]). JMJD2A overexpression may promote transformation by blocking oncogene-induced senescence through transcriptional repression of CHD5 [28]. Direct transcriptional repression of Sp1 by JMJD2A also promotes metastasis of breast cancer [29]. In addition to direct transcriptional regulation, JMJD2A also interacts with transcription factors and functions as either a corepressor or a coactivator. JMJD2A regulates proliferation and apoptosis by functioning as a corepressor for p53 [47]. It also promotes tumor progression by interacting with E2Fs and enhancing the repression of tumor suppressor ARHI expression [48]. In contrast, when interacting with hormone receptors of estrogen (ER) [49] and androgen (AR) [50], JMJD2A functions as a coactivator. Taken together, these reports suggest that JMJD2A appears to regulate cellular function by targeting specific sets of genes through interactions with distinct DNA binding proteins. However, little is known about how JMJD2A is regulated. One recent report showed that expression of JMJD2A is suppressed by tumor suppressor sirt2 [51]. Moreover, as mentioned above, the stability of JMJD2A is regulated by ubiquitination [11,13,39].

In this study, we showed that JMJD2A can be SUMOylated by K-bZIP and it is one of the factors responsible for global SUMO-2/3 enrichment on KSHV genome euchromatin region during viral reactivation. However, our data here demonstrated that SUMOylation is essential for chromatin binding of JMJD2A and established that JMJD2A-K471R may mimic the knockdown of JMJD2A. Consistently, the cell proliferation defect in JMJD2A knockdown SLK and BCBL-1 cells could only be rescued by WT JMJD2A but not its K471R mutant. As an epigenetic regulator capable of regulating genes associated with cancer progression, selective inhibitors for JMJD2A have been developed [52,53]. Discovery of paralog-specific SUMOylation of JMJD2A suggests that the development of SUMOylation specific inhibitors may be a novel avenue for anti-cancer therapy.

## Materials and methods

### Cell culture

The doxycycline (Dox) -inducible TREx-BCBL-1, with Myc-His-tagged K-Rta (TREx-MH-K-Rta BCBL-1), cell line was maintained in RPMI 1640 containing 15% FBS, 20 μg/ml blasticidin and 200 μg/ml hygromycin (Invitrogen, Carlsbad, CA). Latently infected KSHV in TREx-MH-K-Rta BCBL-1 cells were induced with 0.2 μg/ml Dox for viral reactivation. The JMJD2A constitutive knockdown BCBL-1 cell line was generated in a previous study [24]. Cell line TREx-MH-K-Rta-shJMJD2A BCBL-1 was maintained as described for TREx-MH-K-Rta BCBL-1 cells and supplemented with 1 μg/ml puromycin (Invitrogen). JMJD2A knockdown and K-Rta expression was confirmed by immunoblotting analysis. The JMJD2A and K471R overexpression cell lines were generated by transfection of plasmids expressing Flag-JMJD2A-WT shRNA resistant or Flag-JMJD2A-K471R shRNA resistant into TREx-MH-K-Rta-shJMJD2A BCBL-1. Cells were selected for 30 days with 200 μg/ml G418 (AMRESCO) and purified by Ficoll. Expression of Flag-JMJD2A-WT and K471R was tested by Western blot using anti-JMJD2A antibody.

293T cells and SLK cells were maintained in DMEM containing 10% FBS.

### Expression vector

JMJD2A tagged with an N-terminal Flag was expressed from pcDNA3 vector. Mutation of specific lysine residues to arginine was introduced into JMJD2A by site-directed mutagenesis using specific primers listed in S1 Table. T7-SUMO-1, T7-SUMO-2/3, HA-K-bZIP-WT, HA-K-bZIP-L75A and HA-PIAS3 were also expressed from plasmid pcDNA3. Flag-JMJD2A-WT shRNA resistant and Flag-JMJD2A-K471R shRNA resistant were cloned into pLenti4-CMV/TO vector.

### Chromatin Immunoprecipitation-Sequencing (ChIP-Seq) and real-time quantitative PCR (qPCR)

ChIP was performed according to the protocol from Dr. Farnham’s laboratory (http://genomics.ucdavis.edu/farnham). Briefly, chromatin DNA from BCBL-1 cells was harvested after fixation with 1% formaldehyde. Chromatin DNA from 1 x 10^7^ cells was used for each ChIP assay. Anti-JMJD2A rabbit polyclonal antibody, ChIP grade anti-SUMO-2/3 (Abcam, ab81371) mouse monoclonal antibody, ChIP grade anti-H3K9me3 (Abcam, ab8898) rabbit polyclonal antibody and rabbit non-immune serum IgG (Alpha Diagnostic International) were used for the ChIP assays. 50 ng of ChIP’d DNA eluted in 50 μl of ddH_2_O was used for ChIP-seq library preparation, according to the protocol from Illumina. DNA fragment libraries (~400 bp) were analyzed for paired-end sequencing on Illumina HiSeq 2000. The ChIP-Seq data was aligned with the KSHV genome and hg19 build by Partek Genomics Suite (Partek Inc. USA). ChIP DNA was confirmed for successful IP using SYBR Green-Based real-time qPCR analysis by CFX connect real-time PCR detection system (Bio-Rad, Richmond, CA). Specific primer sets were designed to amplify potential binding sites. Primer sequences are listed in S2 Table.

### RNA-seq and reverse transcription-qPCR (RT-qPCR) analysis

Total RNA was prepared from TREx-MH-K-Rta-shJMJD2A, -shJMJD2A-Flag-JMJD2A-WT and -K471R BCBL-1 cell lines with 0 hour and 24 hours Dox treatment using TRIzol (Invitrogen, Carlsbad, CA) according to the manufacturer’s instructions. RNA-seq was performed at the Sequencing Core of National Research Program for Genomic Medicine at the National Yang-Ming University using Illumina HiSeq 2000. Sequencing reads were processed as described previously [21]. In this study, the sequence reads that did not map to hg19 were aligned to the KSHV genome. The transcript frequency was determined in reads per kilobase of transcript per million mapped reads (RPKM) with transcriptome information obtained from Ensembl Release 75 by Partek Genomics Suite. Genes with RPKM > 0.05 were considered as expressed in cells and analyzed for further study. Differential expression of genes in KSHV reactivation at 24 hours verses latency was analyzed by comparing RPKM and calculated as fold change. To compare the expression profile similarity between TREx-MH-K-Rta-shJMJD2A, shJMJD2A-Flag-JMJD2A-WT and -K471R BCBL-1 cell lines, the RPKM in 0 hour and in 24 hours Dox treatment were analyzed for hierarchical clustering by dChip software. The genes used for dChip analysis were listed in S3 and S4 Tables. The biological functions of genes regulated by JMJD2A during KSHV reactivation were analyzed by Ingenuity Pathway Analysis (IPA) software (http://www.ingenuity.com) using IPA spring release 2016. The genes used for IPA analysis were listed in S5 Table. Differential gene expression was analyzed by comparing RPKMs from each sample and verified using real-time RT-qPCR. 0.5 μg of total RNA was reverse-transcribed into cDNA using Oligo-d(T)_18_ and SuperScript III first-strand synthesis system (Invitrogen). qPCR was performed according to the manufacturer's protocol (iQ SYBR Green Supermix, Bio-Rad). Primer sequences are listed in S6 Table.

### Immunofluorescence Assay (IFA)

293T cells were seeded and transfected using TransFectin (Bio-Rad, 170–3351). After 24 hours transfection, cells were reseeded on glass coverslips placed in 6-well plates, and fixed by 4% paraformaldehyde for 20 min. Cells were then washed with PBS in 3 times and permeabilized for 15 min using 0.5% Triton X-100 in PBS. Coverslips were washed 3 times with PBS, incubated in blocking solution (1% BSA in PBS), and further incubated with primary antibodies, anti-JMJD2A antibody and anti-H3K9me3 antibody (Abcam, ab8898) diluted in blocking solution for 16 hours. Coverslips were then washed with PBS 3 times and incubated with secondary antibodies (Abcam, ab181448) diluted in blocking solution for 1 hour. Nuclei were stained with Hoechst 33258 (Invitrogen, H3569) in PBS and washed 3 times with PBS. The cells were mounted on glass slides with mounting solution (20 mM n-propylgallate, 80% Glycerol, 20% 1XPBS). Images were visualized by a Lecia DMI4000B fluorescence microscope and analyzed by MetaMorph (Molecular Devices, Transflour).

### *In vitro* SUMOylation assay and demethylase activity assay by purified proteins

Reactions with purified WT or mutant JMJD2A were incubated with recombinant K-bZIP WT, KbZIP L75A using the SUMOlink SUMO-1 and SUMO-2/3 kit (Active Motif, 40120, 40220) for *in vitro* SUMOylation assays. Reaction products were analyzed by immunoblotting. For demethylase activity assays, purified wild type or mutant JMJD2A were incubated with histone proteins (Sigma-Aldrich, St. Louis, MO, Sigma H9250) for 1 hour at 37°C and reactions were analyzed by immunoblotting.

### *In vivo* SUMOylation assay and demethylase activity assay by 293T cell transfection

293T cells were transfected using transfection reagent, TransFectin for specific protein overexpression. After 48 hours transfection, cells were subjected to immunoprecipitation by anti-Flag M2 beads (Sigma-Aldrich, M8823-1ML) and immunoblotting by specific antibodies. For demethylase activity assay, overexpressed JMJD2A was immunoprecipitated by anti-Flag M2 beads and incubated with histone proteins for 1 hour at 37°C. Reactions were analyzed by immunoblotting using anti-H3K9me3 antibody.

### Immunoprecipitation and immunoblotting

Transfected 293T cells were collected in lysis buffer containing 0.5% NP-40, 1X protease inhibitor cocktail (Roche, 04 693 132 001), and 40 mM N-Ethylmaleimide (NEM, Sigma-Aldrich, E3876-5G). TCLs were incubated with anti-Flag M2 beads for 1 hour at 4°C. Flag-JMJD2A-WT and -K471R complexes were captured by anti-Flag M2 beads. Beads were washed with 0.5% NP-40 for 3 times and the bound proteins were analyzed by immunoblotting. Antibodies used for immunoblotting were anti-α-Tubulin (Sigma-Aldrich, T6074-200UL), anti-PIAS3 (Cell Signaling, D5F9), anti-H3K9me3 (Abcam, ab8898), anti-H3 (GeneTex, GTX122148), anti-SUMO-1 (Abcam, ab32058), anti-SUMO-2/3 (Abcam, ab3742)

### Quantification of KSHV virions by TaqMan qPCR

To evaluate viral production, supernatants from control and 48 hours Dox-induced TREx-MH-K-Rta-shJMJD2A, -shJMJD2A-Flag-JMJD2A-WT and -K471R BCBL-1 cells were collected. KSHV virion DNA purified from collected supernatants was purified by QIAamp MinElute Virus Spin kits as described previously [54]. Virion productivity was determined by real-time qPCR using a TaqMan probe targeting *orf73* (LANA) [55].

### 3-(4,5-Dimethylthiazol-2-yl)-2,5-diphenyltetrazolium bromide (MTT) assay

TREx-MH-K-Rta-shJMJD2A, -shJMJD2A-Flag-JMJD2A-WT and -K471R SLK (2 x 10^3^) and BCBL-1 (5 x 10^3^) cells were seeded into 96-well plates. After 24 hours, the cell viability was examined by MTT assay (Sigma-Aldrich, M5655) for continuous 4 days. A final concentration of 0.5 mg/ml MTT was added and the formazan crystals were solubilized by 10% SDS. The optical density (OD) was determined by a microplate spectrophotometer at a wavelength of 570 and 660 nm.

## Supporting information

S1 Fig Time-course analysis of JMJD2A binding on the KSHV lytic genome. (A) TCLs from TREx-MH-K-Rta-shCtrl BCBL-1 cells treated with 0.2 μg/ml Dox for 12, 24, and 36 hrs were immunoblotted with antibodies as indicated. (B) A JMJD2A ChIP assay was performed with chromatin prepared from cells treated as described in (A). ChIP DNA was quantified by real-time qPCR using primer pairs specific for promoter regions of KSHV *K6*, *PAN*, *K-bZIP*, *Orf52*, *Orf23* and *Orf25*. Data represent mean±SEM. n = 3.
(TIF)

S2 Fig JMJD2A-K463R/K1036R mutant is SUMOylated by SUMO-1 and SUMO-2/3. (A and B) Flag-JMJD2A-K463R/K1036R was expressed in 293T cells as described in Fig 2C. TCLs were immunoblotted with anti-SUMO-1 and anti-SUMO-2/3 antibodies (A). TCLs were IP’d using M2 beads and analyzed by immunoblotting using anti-SUMO-1, anti-SUMO-2/3 and anti-JMJD2A antibodies (B). (C) *in vitro* SUMOylation assay of JMJD2A was performed with the indicated combination of SUMO isoforms and either WT or E3 ligase dead mutant (L75A) of K-bZIP. 4%-20% gradient SDS-PAGE was used to resolve JMJD2A and SUMOylated JMJD2A.
(TIF)

S3 Fig Generation of stable rescue cell lines re-expressing JMJD2A-WT or K471R. (A) Immunoblotting of JMJD2A expression in vector control (pLKO.1) shCtrl, JMJD2A knockdown, and shRNA-resistant JMJD2A-WT and JMJD2A-K471R mutant transfected TREx-MH-K-Rta BCBL-1 cells. (B and C) Successful induction of KSHV K-Rta and K-bZIP expression in JMJD2A-WT and K471R rescue BCBL-1 cells. TCLs isolated from non-induced (0 hr) and 0.2 μg/ml Dox-induced for 24 (B) and 48 (C) hrs TREx-MH-K-Rta-shJMJD2A-Flag-JMJD2A-WT and -K471R BCBL-1 cells were subjected to immunoblotting analysis using antibodies as indicated. Ratio is the relative signal of K-Rta or K-bZIP to α-Tubulin observed for Dox treatment using TREx-MH-K-Rta-shJMJD2A-Flag-JMJD2A-WT BCBL-1 cells as 1.0.
(TIF)

S4 Fig *in vitro* demethylation activity of JMJD2A-WT and -K471R. (A) Purified recombinant JMJD2A-WT and -K471R proteins subjected to *in vitro* demethylation assay using calf thymus histone proteins as substrates. The reaction mixtures were analyzed by immunoblotting using indicated antibodies. (B) 293T cells were transiently transfected with Flag-tag empty vector or Flag-tagged JMJD2A-WT, -K471R or -H188A. *in vitro* demethylation assay was performed using anti-Flag IP’d JMJD2A proteins as in (A).
(TIF)

S5 Fig Histograms show the binding of JMJD2A-WT and -K471R on promoter regions of cancer-related genes. ChIP-seq for JMJD2A was performed using chromatin prepared from TREx-MH-K-Rta-shJMJD2A-Flag-JMJD2A-WT and -K471R BCBL-1 cells. The rectangle indicates the binding pattern of JMJD2A WT (upper panel, red) and K471R (lower panel, orange) on the promoter region (TSS ± 500 bps) of genes as indicated (bottom).
(TIF)

S6 Fig JMJD2A knockdown leads to reduced proliferation of SLK cells. (A) Immunoblot analysis of JMJD2A in SLK cells transduced with lentivirus expressing JMJD2A shRNA. (B) Growth curve of SLK cells infected with lentiviral vectors pLKO.1 and shRNA against JMJD2A.
(TIF)

S7 Fig PIAS3 stimulates JMJD2A modification by SUMO-2/3. (A and B) *in vivo* SUMOylation assays were performed by transfecting 293T cells with plasmids expressing Flag-JMJD2A, T7-SUMO-1 (A) or T7-SUMO-2 and -3 (B) and HA-PIAS3. JMJD2A was IP’d by M2 beads and analyzed by immunoblotting using antibodies as indicated.
(TIF)

S1 Table Primer sequences used for site-directed mutagenesis of JMJD2A SUMOylation mutants. (DOC)

S2 Table Primer sequences used for ChIP-qPCR. (DOC)

S3 Table Cellular gene expression level for dChip analysis. (XLS)

S4 Table KSHV gene expression level for dChip analysis. (XLS)

S5 Table Genes up-regulated in JMJD2A-WT when compared with JMJD2A-K471R rescue BCBL-1 cells after Dox treatment for 24 hours. (XLS)

S6 Table Primer sequences used for RT-qPCR. (DOC)

S7 Table Pathways identified in genes up-regulated by JMJD2A SUMOylation. (XLS)

S8 Table Pathways with more than five molecules up-regulated by JMJD2A SUMOylation. (XLS)
