# Supplementary figures and images for: SUMO modification of a heterochromatin histone demethylase JMJD2A enables viral gene transactivation and viral replication
Source: PLoS Pathog. 2017 Feb 17;13(2):e1006216. doi: 10.1371/journal.ppat.1006216 (PMC5333917; doi:10.1371/journal.ppat.1006216)

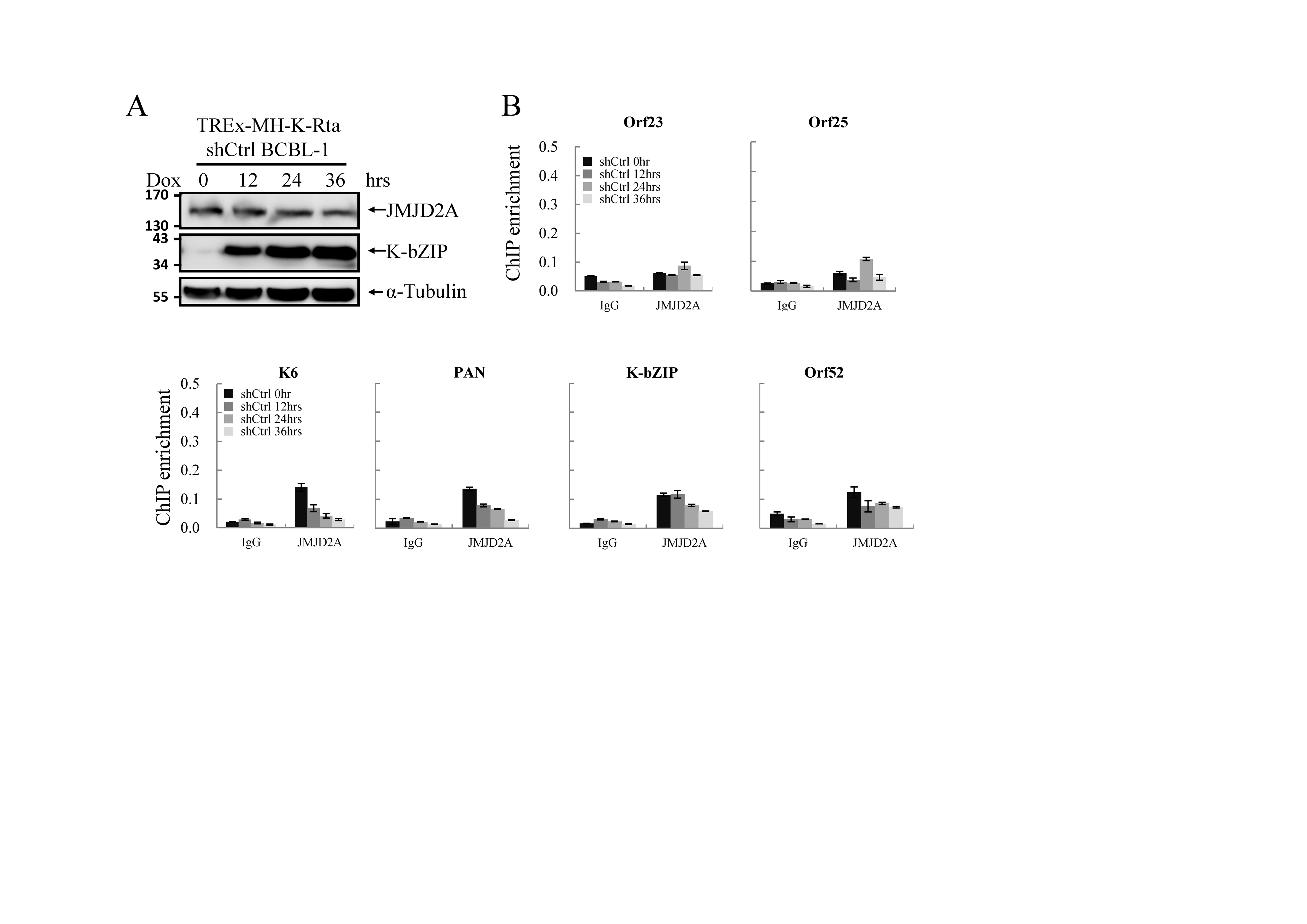

Supplement: S1 Fig — (A) TCLs from TREx-MH-K-Rta-shCtrl BCBL-1 cells treated with 0.2 μg/ml Dox for 12, 24, and 36 hrs were immunoblotted with antibodies as indicated. (B) A JMJD2A ChIP assay was performed with chromatin prepared from cells treated as described in (A). ChIP DNA was quantified by real-time qPCR using primer pairs specific for promoter regions of KSHV K6, PAN, K-bZIP, Orf52, Orf23 and Orf25. Data represent mean±SEM. n = 3. (TIF) [file ppat.1006216.s001.tif]

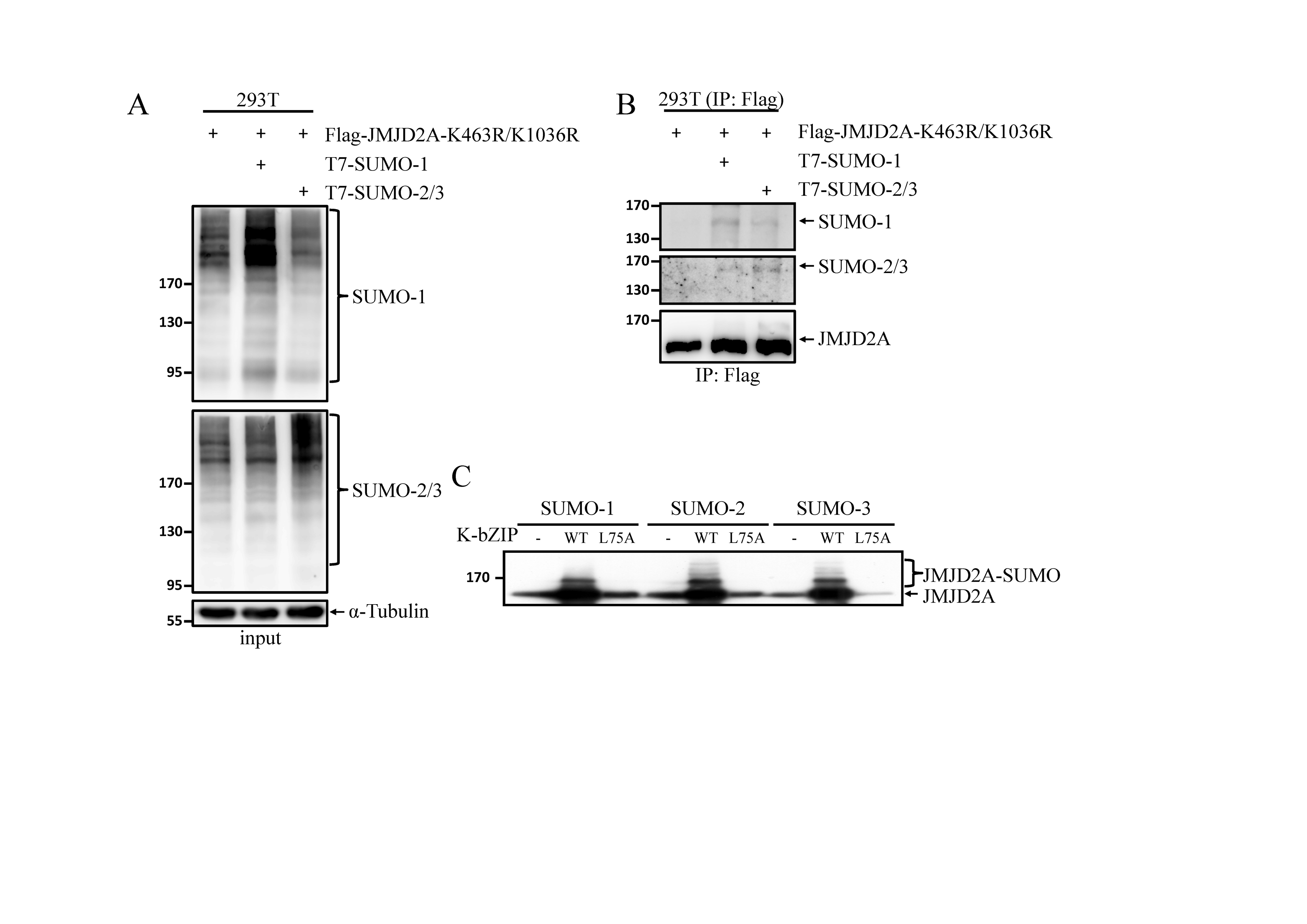

Supplement: S2 Fig — (A and B) Flag-JMJD2A-K463R/K1036R was expressed in 293T cells as described in Fig 2C. TCLs were immunoblotted with anti-SUMO-1 and anti-SUMO-2/3 antibodies (A). TCLs were IP’d using M2 beads and analyzed by immunoblotting using anti-SUMO-1, anti-SUMO-2/3 and anti-JMJD2A antibodies (B). (C) in vitro SUMOylation assay of JMJD2A was performed with the indicated combination of SUMO isoforms and either WT or E3 ligase dead mutant (L75A) of K-bZIP. 4%-20% gradient SDS-PAGE was used to resolve JMJD2A and SUMOylated JMJD2A. (TIF) [file ppat.1006216.s002.tif]

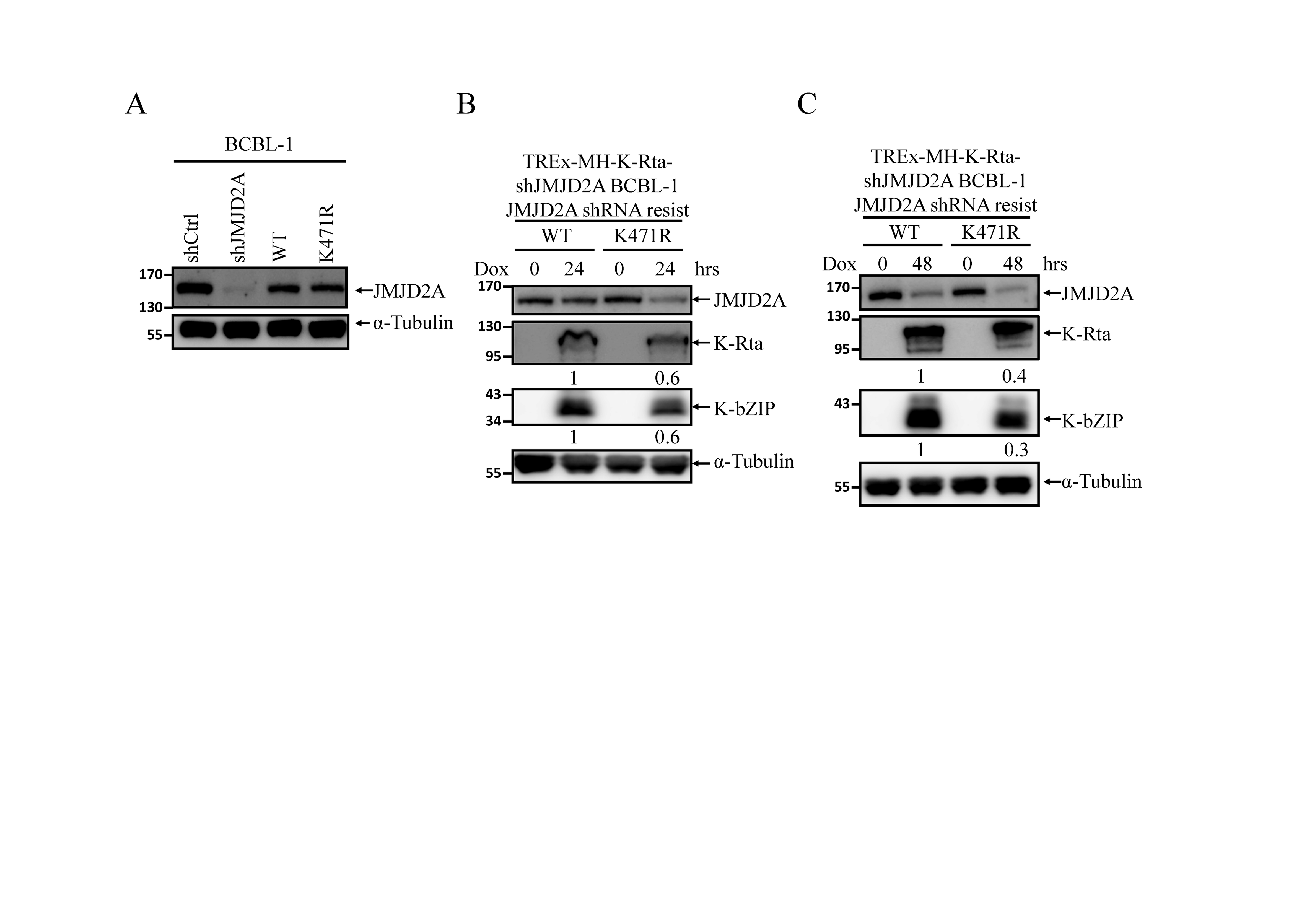

Supplement: S3 Fig — (A) Immunoblotting of JMJD2A expression in vector control (pLKO.1) shCtrl, JMJD2A knockdown, and shRNA-resistant JMJD2A-WT and JMJD2A-K471R mutant transfected TREx-MH-K-Rta BCBL-1 cells. (B and C) Successful induction of KSHV K-Rta and K-bZIP expression in JMJD2A-WT and K471R rescue BCBL-1 cells. TCLs isolated from non-induced (0 hr) and 0.2 μg/ml Dox-induced for 24 (B) and 48 (C) hrs TREx-MH-K-Rta-shJMJD2A-Flag-JMJD2A-WT and -K471R BCBL-1 cells were subjected to immunoblotting analysis using antibodies as indicated. Ratio is the relative signal of K-Rta or K-bZIP to α-Tubulin observed for Dox treatment using TREx-MH-K-Rta-shJMJD2A-Flag-JMJD2A-WT BCBL-1 cells as 1.0. (TIF) [file ppat.1006216.s003.tif]

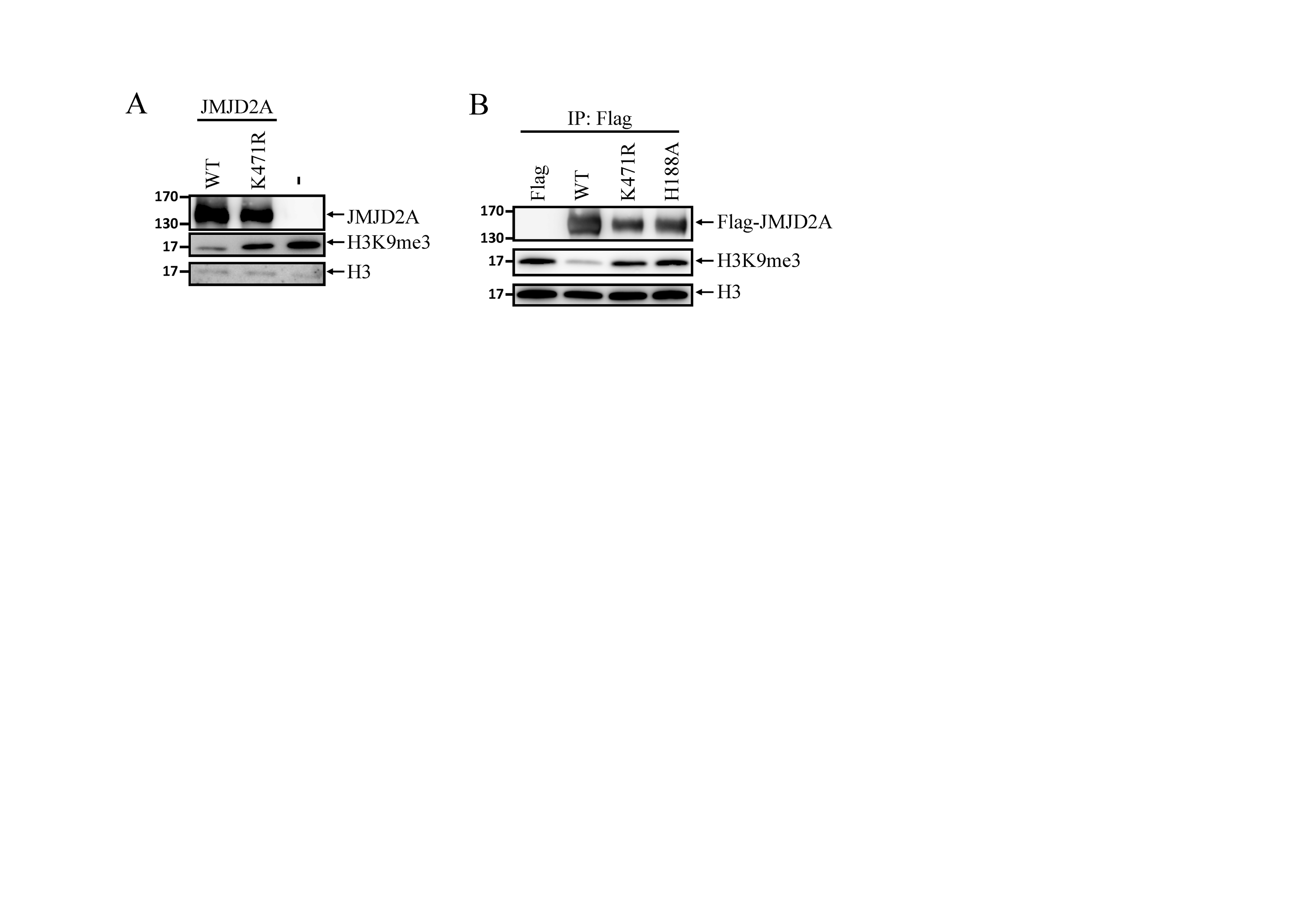

Supplement: S4 Fig — (A) Purified recombinant JMJD2A-WT and -K471R proteins subjected to in vitro demethylation assay using calf thymus histone proteins as substrates. The reaction mixtures were analyzed by immunoblotting using indicated antibodies. (B) 293T cells were transiently transfected with Flag-tag empty vector or Flag-tagged JMJD2A-WT, -K471R or -H188A. in vitro demethylation assay was performed using anti-Flag IP’d JMJD2A proteins as in (A). (TIF) [file ppat.1006216.s004.tif]

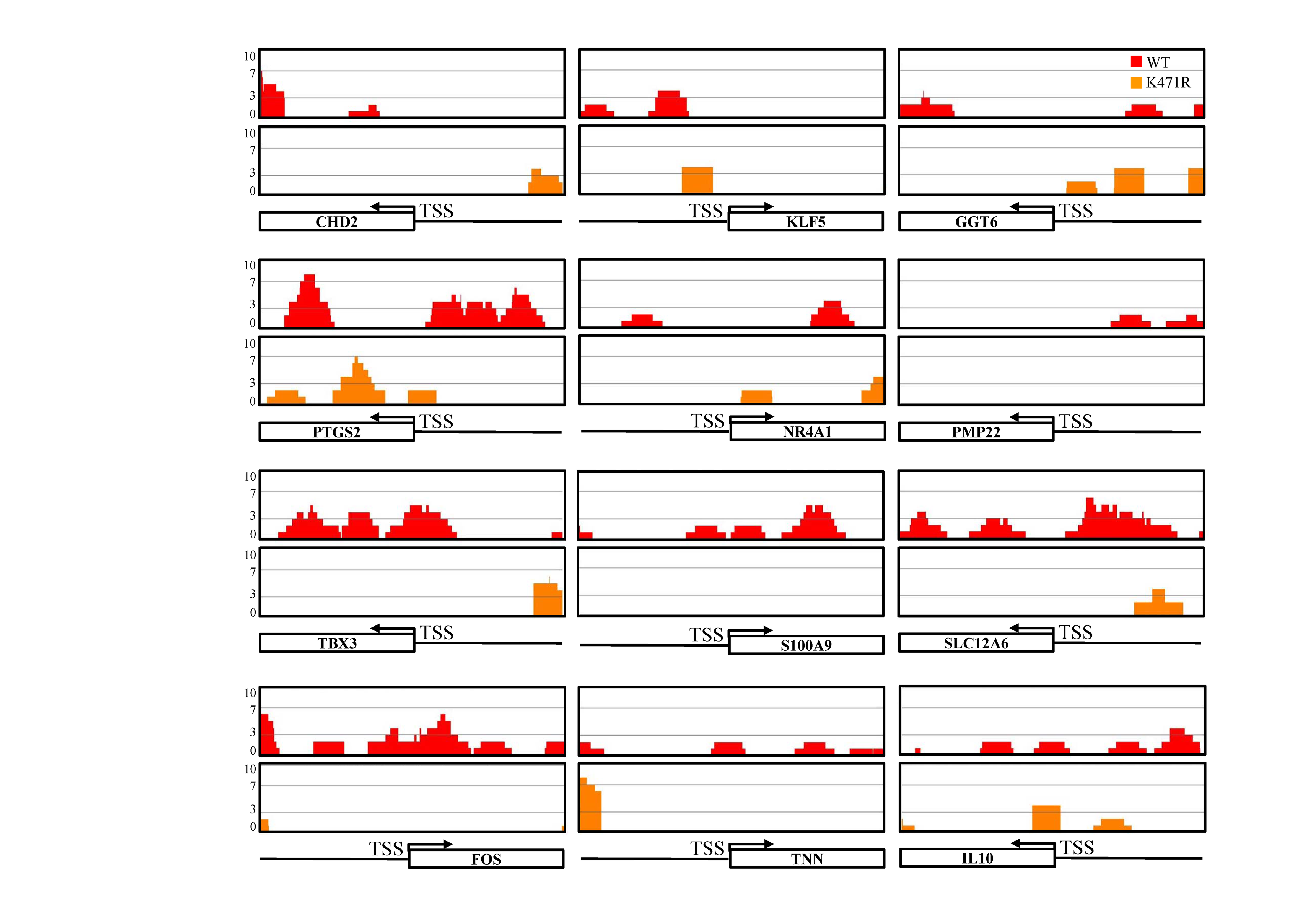

Supplement: S5 Fig — ChIP-seq for JMJD2A was performed using chromatin prepared from TREx-MH-K-Rta-shJMJD2A-Flag-JMJD2A-WT and -K471R BCBL-1 cells. The rectangle indicates the binding pattern of JMJD2A WT (upper panel, red) and K471R (lower panel, orange) on the promoter region (TSS ± 500 bps) of genes as indicated (bottom). (TIF) [file ppat.1006216.s005.tif]

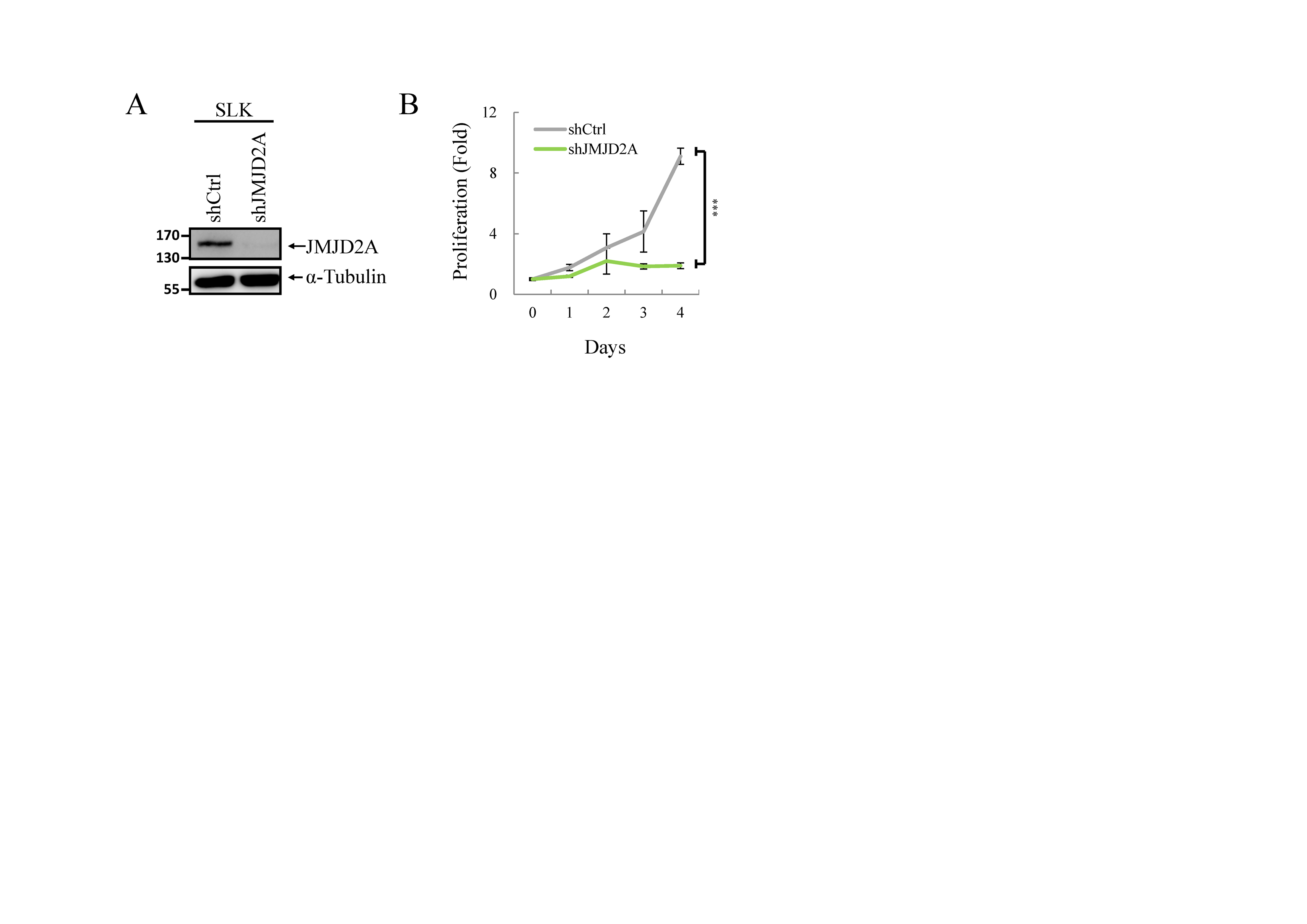

Supplement: S6 Fig — (A) Immunoblot analysis of JMJD2A in SLK cells transduced with lentivirus expressing JMJD2A shRNA. (B) Growth curve of SLK cells infected with lentiviral vectors pLKO.1 and shRNA against JMJD2A. (TIF) [file ppat.1006216.s006.tif]

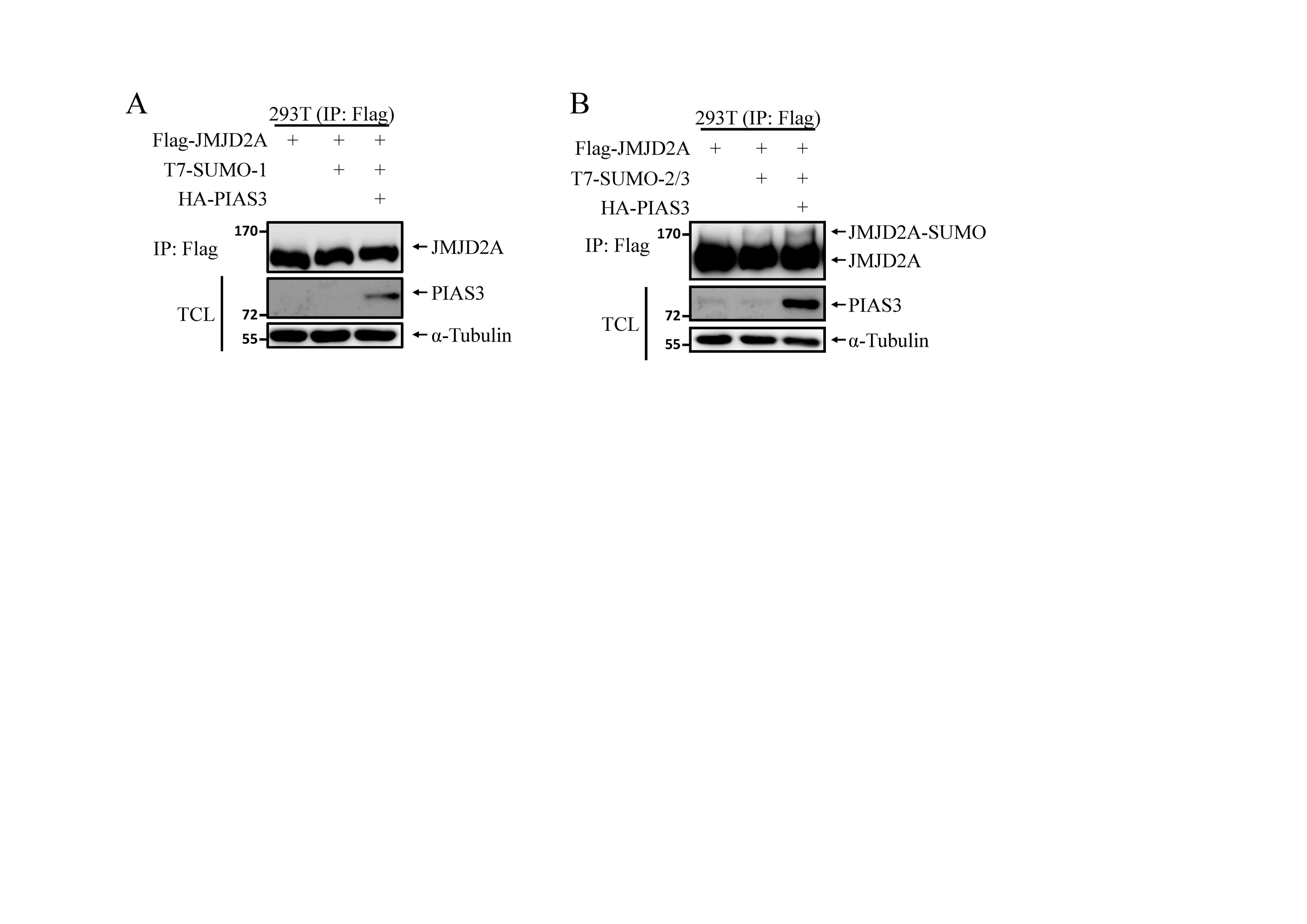

Supplement: S7 Fig — (A and B) in vivo SUMOylation assays were performed by transfecting 293T cells with plasmids expressing Flag-JMJD2A, T7-SUMO-1 (A) or T7-SUMO-2 and -3 (B) and HA-PIAS3. JMJD2A was IP’d by M2 beads and analyzed by immunoblotting using antibodies as indicated. (TIF) [file ppat.1006216.s007.tif]
