# Supplementary material for: SUMO modification of a heterochromatin histone demethylase JMJD2A enables viral gene transactivation and viral replication
Source: PLoS Pathog. 2017 Feb 17;13(2):e1006216. doi: 10.1371/journal.ppat.1006216 (PMC5333917; doi:10.1371/journal.ppat.1006216)
Supplement: S1 Table — (DOC) [file ppat.1006216.s008.doc]

**S1 Table.** Primer sequences used for site-directed mutagenesis of JMJD2A SUMOylation mutants

| Name | Sequence 5’ 🠒 3’ |
| --- | --- |
| K463R-F | TTATTCTGACTCCACTGAAGTCCGATTTGAAGAGCTTAAAAATGTCCGAC |
| K463R-R | GTCGGACATTTTTAAGCTCTTCAAATCGGACTTCAGTGGAGTCAGAATAA |
| K471R-F | AAATTTGAAGAGCTTAAAAATGTCCGACTAGAAGAGGAGGATGAGGAGGA |
| K471R-R | TCCTCCTCATCCTCCTCTTCTAGTCGGACATTTTTAAGCTCTTCAAATTT |
| K1036R-F | GATTTTCACAGAGAAAGAGGTTCGGCAAGAAAAGAAACGGCAACGAGTTA |
| K1036R-R | TAACTCGTTGCCGTTTCTTTTCTTGCCGAACCTCTTTCTCTGTGAAAATC |
