# Supplementary material for: SUMO modification of a heterochromatin histone demethylase JMJD2A enables viral gene transactivation and viral replication
Source: PLoS Pathog. 2017 Feb 17;13(2):e1006216. doi: 10.1371/journal.ppat.1006216 (PMC5333917; doi:10.1371/journal.ppat.1006216)
Supplement: S2 Table — (DOC) [file ppat.1006216.s009.doc]

**S2 Table.** Primer sequences used for ChIP-qPCR

| Name | Sequence 5’ 🠒 3’ |
| --- | --- |
| K6_F | GGTAAAACGTGGCAAGCAGT |
| K6_R | ACCCCAAATAGCCTTTACGG |
| PAN_F | CCGAGTTCCTTATATGCTTACC |
| PAN_F | TCTTAGAAACCCTCACACCT |
| K8_F | CGGCACACTGTACCAGCTGC |
| K8_R | TTCCTCAATGACAGCTTCAT |
| orf52_F | GCGTTAATCAAGGCCCAGAC |
| orf52_R | TGGCCCTGGCTTTATTTGTG |
| orf23_F | AACATAGCTTAGACCAGCGG |
| orf23_R | AAGAAGACCAGGCTAGACGA |
| orf25_F | TGTAGCTGTGCATCTGGG |
| orf25_R | GGATCATGGGAACTTACCGA |
| TBX3_F | GCCGCCTCTAGAATTCAC |
| TBX3_R | AATTGGTCCGAAAGCGTC |
