# Supplementary material for: SUMO modification of a heterochromatin histone demethylase JMJD2A enables viral gene transactivation and viral replication
Source: PLoS Pathog. 2017 Feb 17;13(2):e1006216. doi: 10.1371/journal.ppat.1006216 (PMC5333917; doi:10.1371/journal.ppat.1006216)
Supplement: S6 Table — (DOC) [file ppat.1006216.s013.doc]

**S6 Table.** Primer sequences used for RT-qPCR

| Name | Sequence 5’ 🠒 3’ |
| --- | --- |
| K6_F | CGCCTAATAGCTGCTGCTACGG |
| K6_R | TGCATCAGCTGCCTAACCCAG |
| PAN_F | CATTCGTTGTTTCGGTTCTG |
| PAN_R | CACCATTACAGCACTAGCCT |
| K8_F | GGTCTGTGAAACGGTCATTGA |
| K8_R | TCTATGTAGTCGCCTCTTGGA |
| orf52_F | GGCACCAGGAGGCGGT |
| orf52_R | TCGCTTAGAATCGACGTCTGC |
| orf23_F | TGCCGTCACATATCAGTTCGA |
| orf23_R | CCCCAAAGACCGTCAAAGC |
| orf25_F | CTCGGCGACGTGCTATACAAT |
| orf25_R | TGCCGACAAGGACTGTACATG |
| TBX3_F | GAAGGCGAATGTTTCCTCC |
| TBX3_R | CTTACCAGCCACCATCCA |
